# Putative positive role of inflammatory genes in fat deposition supported by altered gene expression in purified human adipocytes and preadipocytes from lean and obese adipose tissues

**DOI:** 10.1186/s12967-020-02611-6

**Published:** 2020-11-12

**Authors:** Sang-Hyeop Lee, Nak-Hyeon Choi, In-Uk Koh, Bong-Jo Kim, Song Lee, Song-Cheol Kim, Sun Shim Choi

**Affiliations:** 1 Division of Biomedical Convergence, College of Biomedical Science, Institute of Bioscience & Biotechnology, Kangwon National University, Chuncheon, Gangwon-do 24341 Korea; 2 Division of Genome Science, Department of Precision Medicine, Korea National Institute of Health, Korea Disease Control and Prevention Agency, Cheongju-si, Chuncheongbuk-do 28159 Korea; 3 Asan Institute for Life Science, Asan Medical Center, University of Ulsan College of Medicine, 88 Olympic-ro 43-gil, Songpa-gu, Seoul, 05505 Republic of Korea; 4 Division of Hepato-Biliary and Pancreatic Surgery, Department of Surgery, Asan Medical Center, University of Ulsan College of Medicine, 88 Olympic-ro, Songpa-gu, Seoul, 05505 Republic of Korea

**Keywords:** RNA-seq, Adipocytes, Preadipocytes, Inflammatory genes, Visceral adipose tissue

## Abstract

**Background:**

Obesity is a chronic low-grade inflammatory disease that is generally characterized by enhanced inflammation in obese adipose tissue (AT). Here, we investigated alterations in gene expression between lean and obese conditions using mRNA-Seq data derived from human purified adipocytes (ACs) and preadipocytes (preACs).

**Results:**

Total mRNA-seq data were generated with 27 AC and 21 preAC samples purified from human visceral AT collected during resection surgery in cancer patients, where the samples were classified into lean and obese categories by BMI > 25 kg/m^2^. We defined four classes of differentially expressed genes (DEGs) by comparing gene expression between (1) lean and obese ACs, (2) lean and obese preACs, (3) lean ACs and lean preACs, and 4) obese ACs and obese preACs. Based on an analysis of comparison 1, numerous canonical obesity-related genes, particularly inflammatory genes including *IL-6*, *TNF-α* and *IL-1β*, i.e., the genes that are expected to be upregulated in obesity conditions, were found to be expressed at significantly lower levels in obese ACs than in lean ACs. In contrast, some inflammatory genes were found to be expressed at higher levels in obese preACs than lean preACs in the analysis of comparison 2. The analysis of comparisons 3 and 4 showed that inflammatory gene classes were expressed at higher levels in differentiated ACs than undifferentiated preACs under both lean and obese conditions; however, the degree of upregulation was significantly greater for lean than for obese conditions. We validated our observations using previously published microarray transcriptome data deposited in the GEO database (GSE80654).

**Conclusions:**

Taken together, our analyses suggest that inflammatory genes are expressed at lower levels in obese ACs than in lean ACs because lean adipogenesis involves even greater enhancement of inflammatory responses than does obese adipogenesis.

## Background

A widely accepted notion about obesity is that inflammatory responses are elevated in the serum as well as adipose tissue (AT) of obese organisms, as a so-called low-grade inflammatory disease [1]. AT is a primary organ that maintains homeostasis between energy uptake and energy expenditure, in which excess energy is stored in the form of triacylglycerols, whereas free fatty acids are released during fasting [2, 3]. AT is also an endocrine organ that secretes various bioactive factors, namely, adipokines, that regulate the whole-body level of immune and inflammatory responses [4–6]. At the cellular level, obesity is defined as accelerated AT expansion and remodeling that induces either AT hypertrophy (i.e., adipocyte expansion due to excessive fat storage) or hyperplasia (i.e., increased adipogenesis from preadipocytes) through extracellular matrix (ECM) remodeling and angiogenesis [7–9]. Various ECM proteins, including *MMP2, ADAM, TIMP*, *CTSK,* and *CTSS,* are altered in obese AT [7, 10–12]. Angiogenic genes such as *VEGF* and *ANGPT2* are upregulated in response to activated *HIF1A* (i.e., hypoxia-related transcription factor) [13, 14]. *LEP* and *ADIPOQ* are two other genes that have potential adipokine functions involved in AT remodeling [8, 15].

Several studies have shown that the accumulation of excess fat in AT leads to the release of inflammatory mediators, such as *TNF-α* and *IL-6*, and the reduction of anti-inflammatory cytokines, such as adiponectin, is associated with chronic inflammation in obese individuals. It is also known that excess fat that overflows from AT can deposit in other organs such as the liver, pancreas, and muscle, causing insulin resistance [16]. In addition, oxidative stress due to excessive nutrient intake can contribute to increased inflammation associated with obesity [17]. Increased serum levels of C-reactive protein (CRP) are a marker of chronic inflammation in obesity [18]. In fact, numerous studies have argued that the inflammatory responses mounted in AT and the accompanying extensive molecular and cellular changes are responsible for the excess fat deposition associated with the metabolic pathogenicity of obesity such as diabetes and atherosclerosis [19, 20].

In contrast, some recent studies have provided an opposite view of the pathogenic role of the inflammatory response in obesity and obesity-related metabolic diseases [21–24], i.e., a positive role of inflammatory responses in controlling fat deposition. For instance, Ye and McGuinness [21] showed that inflammatory responses are required for the maintenance of a healthy AT microenvironment for AT remodeling and expansion. By constructing three mouse models with adipocyte (AC)-specific attenuated inflammatory responses, Asterholm et al. [22] showed that proper AT remodeling and expansion are executed by inflammatory responses at the level of ACs, whereby reduced or impaired local inflammatory responses in the AC cause pathogenic obesity-related conditions, such as hepatic steatosis or metabolic dysfunction, due to ectopic lipid accumulation [25, 26]. Using a mouse model with AC-specific inflammation inhibition, Zhu et al. [24] showed that suppressing AC inflammation actually promotes rather than reduces insulin resistance in mice. According to Rakotoarivelo et al. [27] the expression of inflammatory cytokines such as *IL-6*, *TNF-α* and *IL-1β* in AT is highly heterogeneous among obesity-associated diabetes patients, and 30% of obese patients do not express most of these inflammatory cytokines in the AT. Haffa et al. [28] showed that these cytokines do not show obesity-related gene expression levels in AT. Baranova et al. [29] reported a paradoxical decrease in the expression of pro-angiogenic genes, which is another gene class that is expected to increase in obese individuals. Moreover, a recent AT-derived RNA-seq analysis of pigs showed that pigs with thicker back fat tend to express significantly lower levels of immune and inflammatory genes than pigs with thinner back fat, indicating that the expression of high inflammatory genes may be associated with lower fat accumulation in AT [30].

We think that the contradictory conclusions regarding the positive or pathogenic role of inflammation in obesity are largely due to the use of differential experimental designs among different studies. As expected, retrieving lean (or healthy) AT samples is much more difficult than retrieving obese AT samples in humans. Purification of cells such as ACs, preadipocytes (preACs), macrophages, and endothelial cells residing in AT is even more difficult. For this reason, a few studies have aimed to identify differentially expressed genes (DEGs) by directly comparing lean and obese ATs from cohorts of lean and obese individuals [9, 31–33]. Instead, some studies have investigated DEGs in AT samples during weight loss induced by bariatric surgery for the same individuals [34–36], and others have analyzed DEGs between subcutaneous adipose tissue (SAT) and visceral adipose tissue (VAT) derived from the same obese individuals [37, 38]. Gene expression has rarely been analyzed between obese and lean ATs even in model organisms such as mice, where a few studies have pursued identification of DEGs between mice fed normal chow and mice fed a high-fat diet (HFD) [22, 24, 39]. A consistent conclusion from these transcriptome-based analyses is that inflammatory genes in ATs are decreased as organisms lose weight and increased as organisms become obese, which is the basis for understanding the relationship between inflammatory genes and obesity to date. However, most of these experimental designs were unable to take into account the fat deposition process in AT under both lean conditions and obese conditions.

Here, we try to answer which of these two scenarios, i.e., the pathogenic role or the positive role of inflammatory genes in fat deposition, better explains the role of inflammatory genes in obesity by analyzing mRNA-Seq data generated from highly purified ACs and preACs obtained from visceral (omental) AT (i.e., VAT) collected during resection surgery in cancer patients. Note that these cancer patients were all Koreans who had undergone resection surgery at a single hospital, Asan Medical Center in Seoul, Korea.

## Results

### Four-way identification of DEGs

**Fig. 1:**
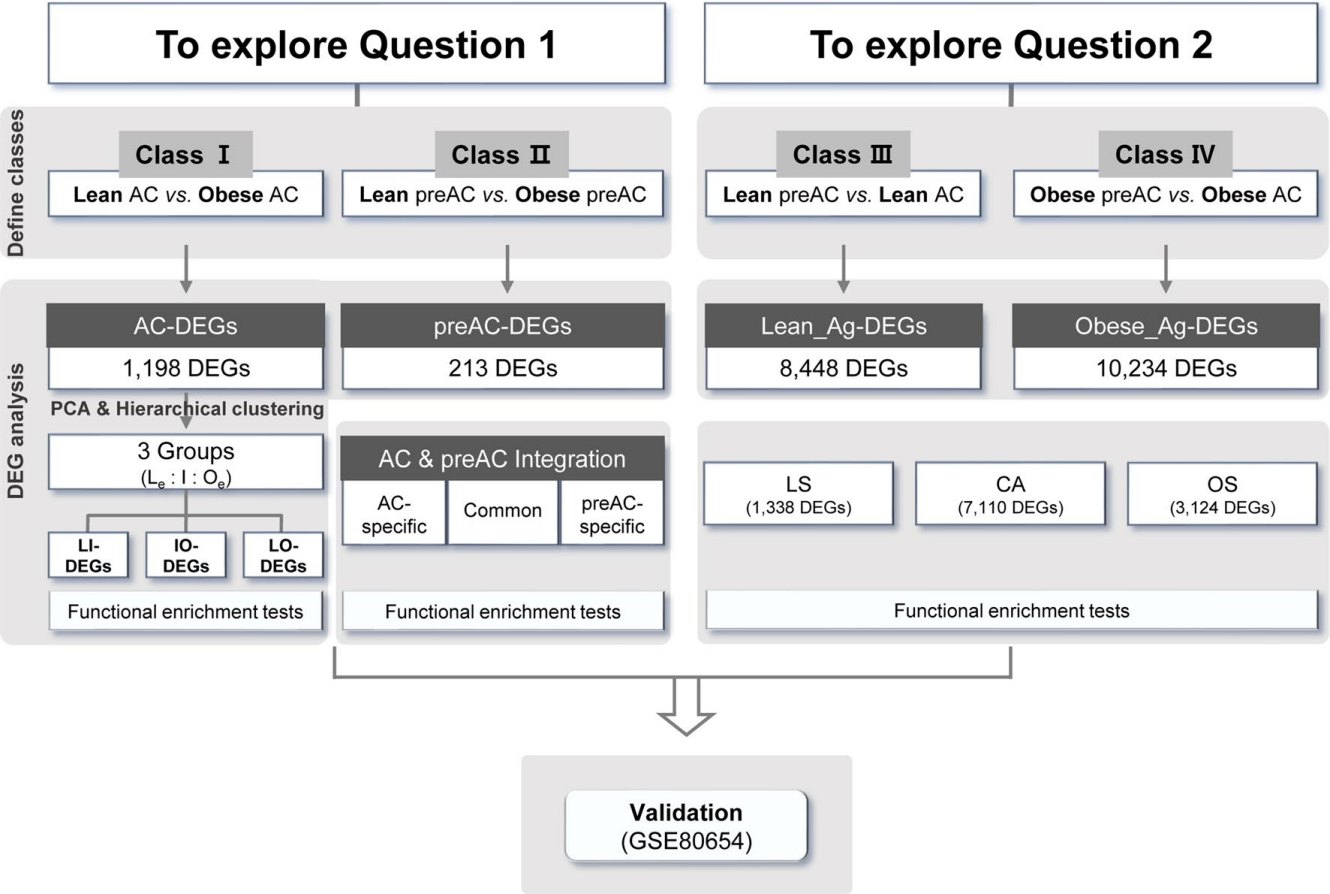
Overall schematic of the workflow. Workflow is depicted as a flowchart. Abbreviations used in this flowchart are as follows; AC, adipocyte; preAC, preadipocyte; DEG, differentially expressed gene; L_e_, lean extreme; O_e_, obese extreme; Ag, AC differentiation; LS, lean AC differentiation-specific; OS, obese AC differentiation-specific; CA, commonly altered for both

Figure 1 shows a flowchart of the study procedure. After producing mRNA-seq data from the purified ACs and preACs of lean and obese individuals, we investigated two different questions: (1) which genes are significantly upregulated or downregulated under obese conditions in comparison with lean conditions, and (2) which alterations in gene expression detected for obese adipogenesis (i.e., the information extracted from the DEGs obtained by comparing ACs and preACs from obese individuals) are significantly different from those detected for lean adipogenesis (i.e., the information extracted from the DEGs obtained by comparing ACs and preACs from lean individuals). As a validation, we compared our conclusion with that of a previously published paper that provided a list of DEGs obtained by comparing gene expression between lean and obese ACs derived from lean and obese SAT samples; the original dataset produced by the microarray platform was downloaded from the GEO database (GSE80654). How similar that list of DEGs is to our results discussed later in "Discussion" section.

We decided to estimate four different classes of DEGs from four different types of mRNA-Seq data, i.e., ‘lean AC’ (L-AC), ‘obese AC’ (O-AC), ‘lean preAC’ (L-preAC), and ‘obese preAC’ (O-preAC) (Additional file 1: Figure S1): ‘Class I: AC-DEGs’ from the comparison of expression between ‘L-AC’ and ‘O-AC’, ‘Class II: preAC-DEGs’ between ‘L-preAC’ and ‘O-preAC’, ‘Class III: Lean_Ag-DEGs’ between ‘L-preAC’ and ‘L-AC’, and ‘Class IV: Obese_Ag-DEGs’ between ‘O-preAC’ and ‘O-AC’. Various thresholds were tested to select DEGs (Additional file 2: Table S1), and DEGs were identified for the four classes mentioned above. Class I and II DEGs were investigated to answer question #1 described above, i.e., to determine the differences between obese ACs and lean ACs and between obese preACs and lean preACs. Class III and IV DEGs were chosen to answer question #2 described above, i.e., to determine how gene expression is altered during obese and lean AC adipogenesis.

### Defining intermediate obesity samples in ‘Class I: AC-DEGs’

**Fig. 2:**
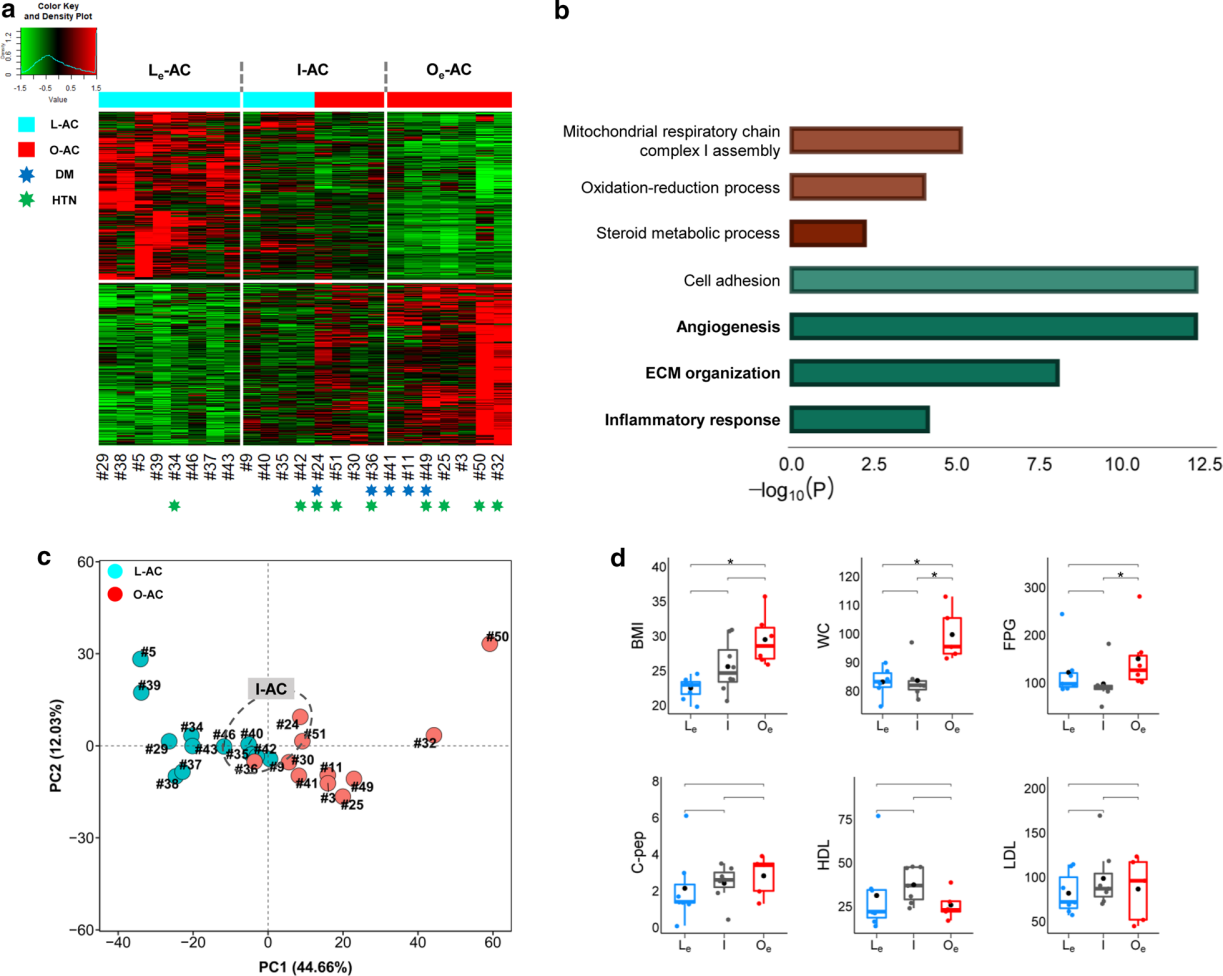
Classification of AC samples by DEGs. **a** Construction of the heatmap accompanied by unsupervised hierarchical clustering. Heatmap coupled with unsupervised hierarchical clustering is generated by the 1,198 DEGs that are estimated by comparing mRNA expression with *P* < 0.01 between 12 L-AC and 11 O-AC samples. Cyan and red bars located at top of the heatmap are replaced for the dendrograms generated by the clustering procedure where cyan and red bars represent lean and obese samples, respectively. ‘L_e_’, ‘I’, and ‘O_e_’ represent extreme, intermediate, and obese extremes, respectively, as explained in the main text. The samples in blue and green ‘*’ indicate whether the patients have been treated with drugs for diabetes (DM) or hypertension (HTN), respectively. **b** GO analysis of ‘AC-DEGs’; the red and green bars represent upregulated (i.e., genes with higher levels in obese ACs than in lean ACs) and downregulated genes (i.e., genes with lower levels in obese ACs than in lean ACs), respectively. The functional terms with top significance by sorting *P* values in the GO analysis are represented only in the graph. The scale on the bottom indicates the -log_10_ *P* value of the significance of enrichment of each functional term. **c** PCA analysis. PCA analysis is performed with ‘Class I: AC-DEGs’. The emergence of ‘I-AC’ samples identified in (**a**) is confirmed as the samples in the group are located in the middle of the plot marked with dotted circles. **d** Box plot analysis of selected clinical information among ‘L_e-_AC’, ‘I-AC’, and ‘O_e_-AC’ samples; BMI, WC, FPG, C-pep, LDL, and HDL were selected. The statistical test of clinical information among the groups was performed by the ‘Wilcoxon rank-sum test’. ‘*’ indicates that the statistical test is *P* < 0.05

A total of 1,198 genes and 314 genes were classified as ‘Class I: AC-DEGs’ with *P* < 0.01 and Q < 0.05, respectively (Additional file 2: Table S1). ‘L-AC’ samples could not be differentiated from ‘O-AC’ samples by either of these DEGs in the analysis of unsupervised clustering processed with a heatmap; the samples in the middle of the heatmap did not show gene expression patterns pertinent to the ‘L-AC’ and ‘O-AC’ categories (Fig. 2a). Notably, gene ontology (GO) analysis showed that canonical obesity-related genes involved in inflammation and ECM were downregulated in obese ACs rather than in lean ACs (Fig. 2b). The ambiguity of sample classification by these DEGs was also confirmed in principal component analysis (PCA) (Fig. 2c). Thus, we subcategorized these ambiguous samples separately into the ‘intermediate (I-AC)’ group (#9, 24, 30, 35, 36, 40, 42, 51). The remaining two extreme samples were then named lean-extreme (‘L_e_-AC’) and obese-extreme (‘O_e_-AC’), which were ultimately categorized as 8 ‘L_e_-AC’, 8 ‘I-AC’, and 7 ‘O_e_-AC’, as indicated on top of the heatmap (Additional file 3: Table S2).

Subsequently, for these redefined three groups of samples, some obesity-related clinical information was investigated, including BMI, waist circumference (WC), fasting plasma glucose (FPG) level, C-peptide (C-pep), high-density lipoprotein (HDL) level, and low-density lipoprotein (LDL) level (Fig. 2d). Interestingly, ‘I-AC’ samples were special in that the BMIs of ‘I-AC’ were expectedly positioned between ‘L_e_-AC’ and ‘O_e_-AC’; however, the WC and FPG level of the ‘I-AC’ samples were comparable to those of ‘L_e_-AC’. In addition, except for BMI, WC, and FPG, all the other clinical levels showed no significant difference among these three groups, although ‘I-AC’ was located between ‘L_e_-AC’ and ‘O_e_-AC’. Notably, FPG levels seemed to be associated with WC rather than with BMI.

The existence of a third group, ‘I-AC’ samples, may not be surprising, considering the complexities of molecular etiologies causing obesity involved with various genetic and epigenetic alterations and the unclear association between obesity and obesity-related metabolic diseases.

### Inflammatory genes are expressed at lower levels in obese ACs than in lean ACs

**Fig. 3:**
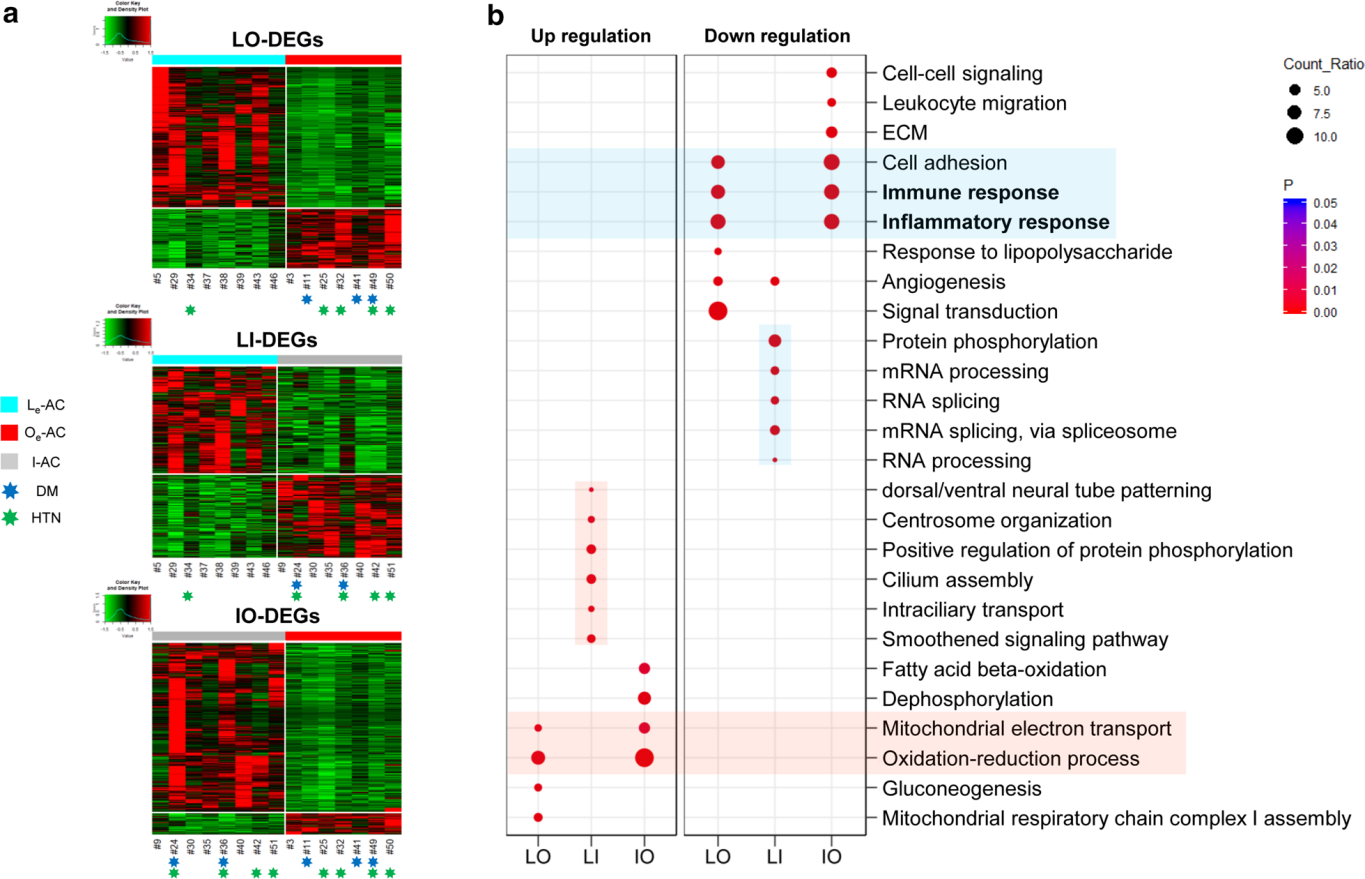
Three classes of DEGs using the three redefined groups of samples. **a** Construction of heatmap coupled with unsupervised hierarchical clustering for three different classes of DEGs. A total of 2,657 ‘LO-DEGs’ (Q < 0.01), 1,474 ‘LI-DEGs’ (P < 0.01), and 1,324 ‘IO-DEGs’ (Q < 0.05) are used to construct a heatmap coupled with unsupervised clustering (refer to Additional file 4: Table S3). The same notations used for Fig. 2a are also used in this heatmap (refer to Fig. 2a legend). **b** Analysis of GO functional terms for each of the three classes of DEGs. ‘LO’, ‘LI’, and ‘IO’ represent ‘LO-DEGs’, ‘LI-DEGs’ and ‘IO-DEGs’, respectively. For each class of DEGs, genes are divided into upregulated genes (i.e., genes with higher levels in obese ACs than in lean ACs) and downregulated genes (i.e., genes with lower levels in obese ACs than in lean ACs). Wide red and blue boxes within the plot indicate upregulated and downregulated genes, respectively, for both ‘LO-DEGs’ and ‘IO-DEGs’. Narrow red and blue boxes within the plot indicate upregulated and downregulated genes, respectively, for the ‘LI-DEGs’

DEGs were re-estimated involving the ‘I-AC’ in three different comparison sets, between ‘L_e_-AC’ and ‘O_e_-AC’ (named ‘LO-DEGs’), between ‘L_e_-AC’ and ‘I-AC’ (named ‘LI-DEGs’), and between ‘I-AC’ and ‘O_e_-AC’ (named ‘IO-DEGs’), with various thresholds (Additional file 4: Table S3). To understand alterations in gene expression related to obesity, we applied different thresholds to produce similar numbers of DEGs for these three categories (indicated ‘*’ in Additional file 4: Table S3). Consequently, a total of 2,657 (Q < 0.01), 1,474 (P < 0.01), and 1,324 (Q < 0.05) DEGs were selected for ‘LO-DEGs’, ‘LI-DEGs’, and ‘IO-DEGs’, respectively; the heatmap of each group was constructed to visualize gene expression differences (Fig. 3a). Notably, the highest number of genes was allocated in ‘LO-DEGs’, despite the stringent threshold applied. Note that we focused on collecting similar numbers of DEGs rather than on determining a single criterion or a single most important gene (although all the thresholds were chosen in ranges considered statistically significant) to reveal trends in gene expressions between two different conditions.

A striking observation emerged from GO analysis. Specifically, a total of 1,874 genes of the 2,657 ‘LO-DEGs’ (70.5%), i.e., genes largely assigned to the inflammatory response and cell adhesion, were expressed at significantly lower levels in ‘O-AC’ than in ‘L-AC’ (Fig. 3b, in the wide blue box). A similar result was observed in Fig. 2b, and the downregulation of these genes seems to be amplified when ‘I-AC’ samples are excluded from the DEG analysis. Moreover, ‘IO-DEGs’ revealed the same pattern as did ‘LO-DEGs’ (Fig. 3b, in the wide blue box). This observation is striking because these classes of genes are all canonical obesity-related genes that are known to be expressed at higher levels in obese AT [19, 20].

By contrast, other DEGs involved in fat metabolism in ‘LO-DEGs’ and ‘IO-DEGs’ were consistent with the previous findings, i.e., upregulation under obese conditions. For instance, a total of 783 genes of the 2,657 ‘LO-DEGs’ (29.5%), including *LEP*, *CES1*, and *NQO1*, that were expressed at higher levels in ‘O_e_-AC’ than in ‘L_e_-AC’, were largely assigned to mitochondrial metabolism and the oxidation–reduction process (ROS) (Fig. 3b, in the wide red box).

‘LI-DEGs’ were also assigned to distinctive GO functions; RNA metabolism genes were expressed at lower levels in ‘I-AC’ than in ‘L_e_-AC’ (i.e., downregulated; narrow blue box in Fig. 3b, and genes involved in centrosome organization and protein phosphorylation were expressed at higher levels in ‘I-AC’ than in ‘L_e_-AC’ (i.e., upregulated; narrow red box in Fig. 3b), confirming that ‘I-AC’ is distinct and not comparable to ‘L_e_-AC’ or ‘O_e_-AC’.

Gene set enrichment analysis (GSEA), i.e., a tool designed to see whether an a priori defined set of genes shows significant differences in expression between two different biological conditions, led to the same conclusion as did GO analysis. Specifically, genes belonging to inflammatory or angiogenesis functions were significantly upregulated in lean ACs rather than obese ACs, whereas genes belonging to cellular respiration functions were significantly upregulated in obese ACs (Additional file 5: Figure S2).

### Detailed examination of expression alterations between lean and obese ACs

**Fig. 4:**
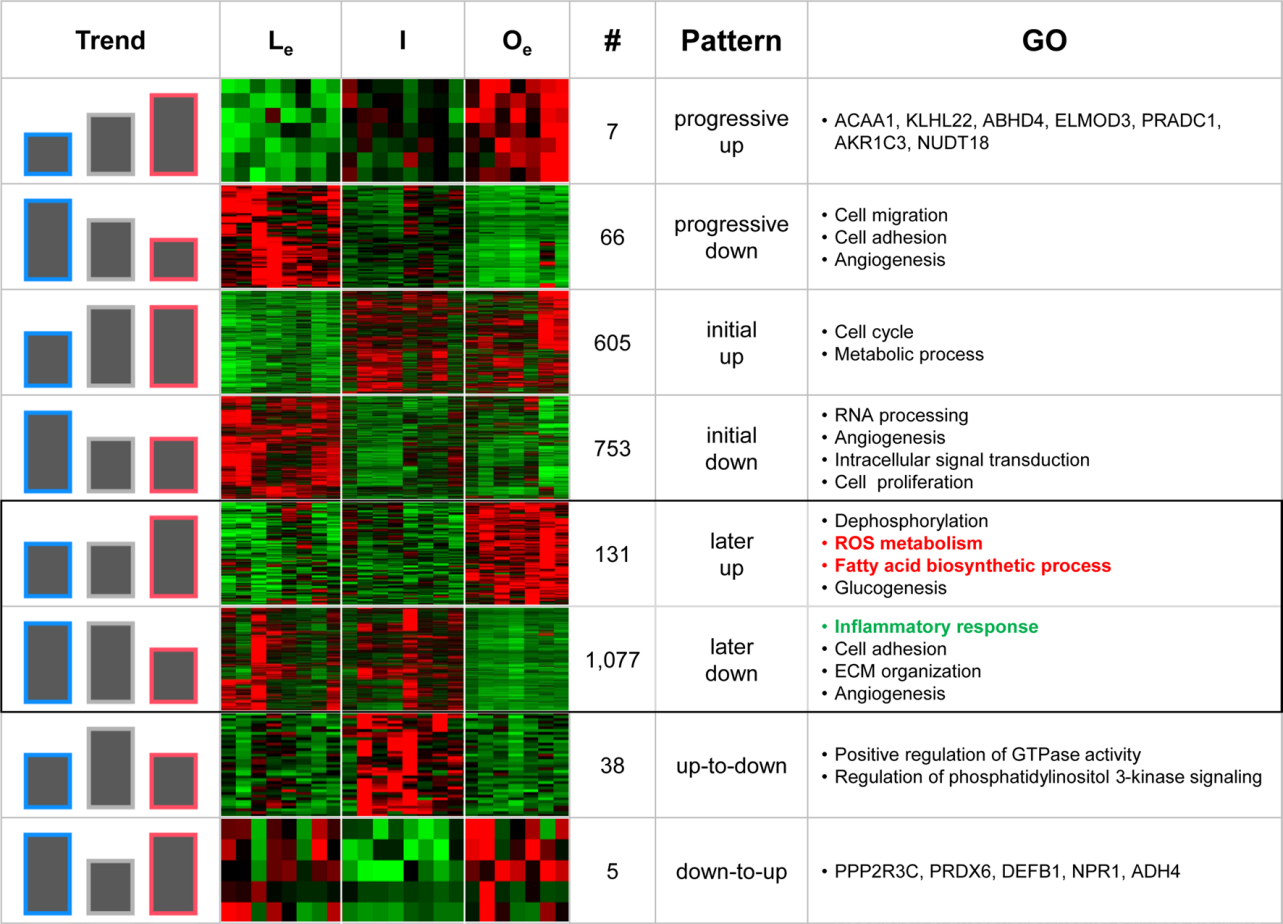
Examination of changes in gene expression between lean ACs and obese ACs. Changes in gene expression are allocated into eight different categories: ‘progressive-up/down’, ‘initial-up/down’ ‘later-up/down’, and ‘up-to-down/down-to-up’. Each category of DEGs is obtained by intersecting ‘LI-DEGs’ and ‘IO-DEGs’ (refer to Additional file 6: Figure S3 for the detailed strategy for the subcategorization). A heatmap is generated for each category of DEGs with genes assigned to each category. The bars in the first column represent the trend of gene expression levels in each category, and the blue, gray, and red lines surrounding each bar represent genes in the L_e_, I, and O_e_ categories, respectively

The regrouped ‘L_e_-AC’, ‘I-AC’, and ‘O_e_-AC’ were assumed to reflect different degrees of obesity based on BMI, as shown in Fig. 2d. Thus, changes in gene expression can be examined further by investigating ‘LI-DEGs’ and ‘IO-DEGs’. For instance, if a gene that is upregulated both in ‘LI-DEGs (i.e., genes expressed at higher levels in ‘I-AC’ than in ‘L_e_-AC’) and ‘IO-DEGs (i.e., genes expressed at higher levels in ‘O_e_-AC’ than in ‘I-AC’), it can be concluded that the gene is ‘progressively upregulated (named ‘progressive-up’) because it is upregulated in ‘L_e_’ compared with that in ‘I’ and upregulated again in ‘I’ compared with that in ‘O_e_’ (refer to Additional file 6: Figure S3). Similarly, if a gene is downregulated in both ‘LI-DEGs’ and ‘IO-DEGs’, the gene is defined as progressively downregulated (named ‘progressive-down’). Using this scheme, genes were allocated into eight different categories as shown in Fig. 4 and Additional file 6: Figure S3. As a result, only seven genes, including *ACAA1, KLHL22, and AKR1C3,* were identified as ‘progressive-up’, while a total of 66 genes were categorized as ‘progressive-down’. Notably, genes assigned to cell migration, cell adhesion, and angiogenesis were allocated to the ‘progressive-down’ category.

Cell cycle and metabolic process genes were upregulated in ‘I-AC’ compared with those in ‘L-AC’ and sustained their expression in ‘O-AC’, i.e., upregulated genes in the ‘LI-DEGs’ category but not in the ‘IO-DEGs’ category (named ‘initial-up’). By contrast, RNA processing, angiogenesis, and signal transduction genes were downregulated in ‘I-AC’ and sustained their expression in ‘O-AC’, i.e., downregulated genes in the ‘LI-DEGs’ category but not in the ‘IO-DEGs’ category (named ‘initial-down’) (Fig. 4). This result indicates that angiogenesis alteration and cell proliferation may start in the early stage of obesity. Genes involved in ROS metabolism and fatty acid biosynthetic process genes were upregulated in the later stage in obesity, i.e., genes not in the ‘LI-DEGs’ category but upregulated in the ‘IO-DEGs’ category (named ‘later-up’). By contrast, genes involved in inflammation, cell adhesion, and ECM organization were downregulated in a later stage in obesity, i.e., genes not in the ‘LI-DEGs’ category but downregulated in the ‘IO-DEGs’ category (named ‘later-down’) (Fig. 4). Notably, genes in the ‘later-down’ category showed that most canonical obesity genes were downregulated in in the later stage of obesity.

### Comparison of gene expression profiles between L-preACs and O-preACs

**Fig. 5:**
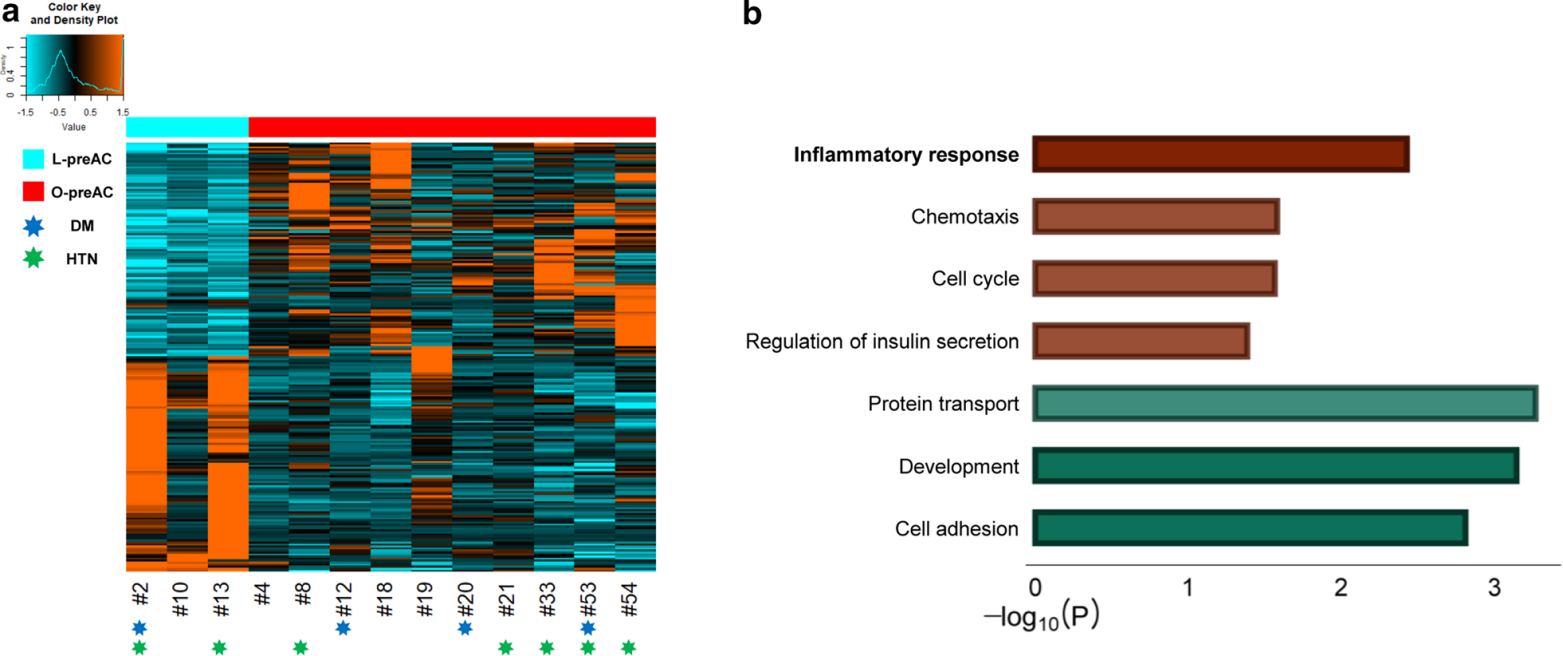
Differentiation of lean preAC samples and obese preAC samples by ‘Class II: preAC-DEGs’. **a** Construction of heatmap accompanied with unsupervised hierarchical clustering. A heatmap coupled with unsupervised hierarchical clustering is generated with a total of 213 ‘Class II: preAC-DEGs’ (P < 0.01) (Additional file 2: Table S1). Refer to the notations in the Fig. 2a legend. **b** GO analysis of ‘preAC-DEGs’; red and green bars represent upregulated (i.e., genes with higher levels in obese preACs than in lean preACs) and downregulated genes (i.e., genes with lower levels in obese preACs than in lean preACs), respectively. Refer to the Fig. 2b legend for how to select the functional terms and the meaning of the scale on the bottom

We obtained a total of 213 ‘Class II: preAC-DEGs’ estimated from 3 ‘L-preACs’ and 10 ‘O-preACs’ (Additional file 2: Table S1, Additional file 3: Table S2), as shown in a heatmap (Fig. 5a). The expression differences were somewhat vague between L-preACs and O-preACs (Fig. 5a). Unlike ‘Class I: AC-DEGs’ in Fig. 2, unsupervised clustering and PCA analysis produced a distinctive classification between ‘L-preAC’ and ‘O-preAC’ (Additional file 7: Figure S4), mainly due to the smaller sample size.

Remarkably, GO analysis of ‘Class II: preAC-DEGs’ revealed the opposite direction for some gene expression levels compared to those observed for ‘Class I: AC-DEGs’. Inflammatory response and chemotaxis genes, i.e., genes that were downregulated in AC comparisons, were upregulated in obese samples compared with lean samples (Fig. 5b). Interestingly, a similar inverse profile between AC and preAC has been reported for miRNAs [40], implicating crosstalk between AC and preAC during AC differentiation. However, cell adhesion genes were significantly downregulated in obese samples, consistent with the direction found in AC comparisons.

### Both lean and obese adipogenesis are required for the enhancement of inflammatory genes

Comparing expression between preACs and ACs was assumed to reveal the changes in gene expression during the process of AC differentiation from preACs (i.e., during adipogenesis). Under this assumption, we investigated how obese AC adipogenesis is differentiated from lean AC adipogenesis by obtaining Class III and Class IV DEGs; ‘Class III: Lean_Ag-DEGs’ were estimated by comparing ‘L-preAC’ and ‘L_e_-AC’, and ‘Class IV: Obese_Ag-DEGs’ were obtained by comparing ‘O-preAC’ and ‘O_e_-AC’ (Additional file 1: Figure S1 and Additional file 2: Table S1). For the two classes, ‘upregulation’ or ‘downregulation’ was determined based on the expression between ACs and preACs, i.e., genes that were expressed at higher levels in ACs than in preACs were upregulated genes, and genes that were expressed at lower levels in ACs than in preACs were downregulated genes.

**Fig. 6:**
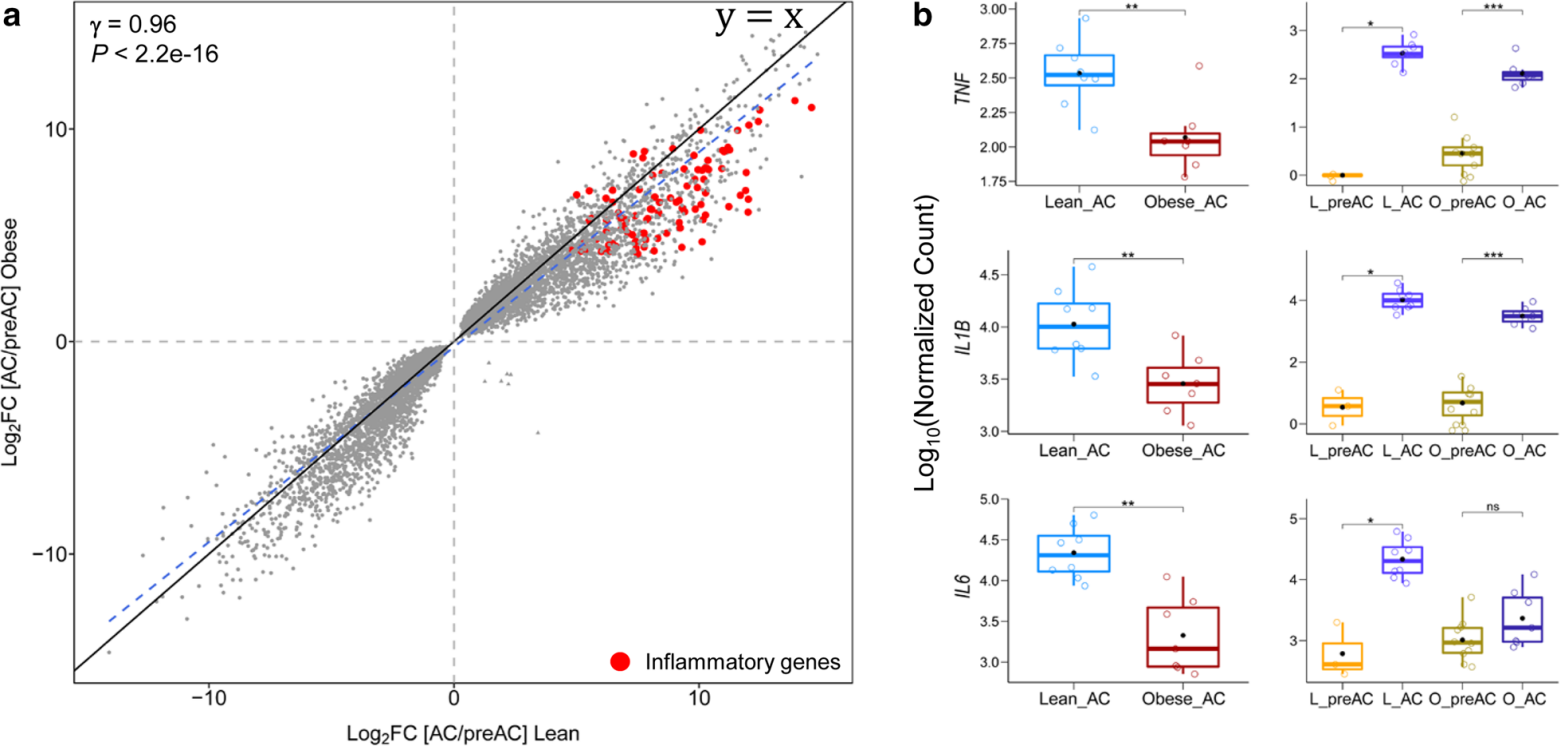
Comparison of inflammatory gene expression in lean and obese adipogenesis. **a** A scatter plot was constructed with a total of 7,118 genes commonly altered in lean and obese adipogenesis (i.e., common DEGs between ‘Lean_Ag-DEGs’ and ‘Obese_Ag-DEGs’). *Log*_*2*_*FC* values of gene expression in ‘Lean_Ag-DEGs’ were plotted against *log*_*2*_*FC* values of gene expression in ‘Obese_Ag-DEGs’. The gray and red dots represent the total 7118 common DEGs and the inflammatory genes, respectively. The blue dashed diagonal line indicates a regression line for the values, while the black diagonal line indicates the line of equality (y = x). The correlation coefficient (γ) was calculated by Pearson correlation test. **b** Boxplots of gene expression values for three selected genes, i.e., *IL-6, TNF-α, and IL-1β* were constructed; the box plots in the left panel provide a comparison of the expression of these three genes in lean and obese AC samples, while the box plots in the right panel provide a comparison between ‘[AC/preAC]_lean’ (i.e., expression levels of these three genes in AC and preAC samples derived from the lean adipogenesis condition) and ‘[AC/preAC]_obese’ (i.e., expression levels of these three genes in AC and preAC samples derived from the obese adipogenesis condition). The statistical test was performed by the ‘Wilcoxon rank-sum test’. ‘*’, ‘**’, and ‘***’ indicate *P* < 0.05, *P* < 0.01, and *P* < 0.001, respectively, while ‘ns’ indicates *P* > 0.05

We found that alterations in gene expression between preACs and ACs measured in the lean condition (i.e., ‘Lean_Ag-DEGs’) was significantly positively correlated with alterations in gene expression between preACs and ACs measured in the obese condition (i.e., ‘Obese_Ag-DEGs’) (γ = 0.96 and P < 2.2e−16, Pearson correlation) (Fig. 6a), indicating that AC differentiation from preACs to ACs involves similar gene expression alterations for both the lean and obese conditions. The degree of alterations in gene expression between preACs and ACs was extremely large, regardless of whether lean or obese samples were assessed; a total of 8,448 genes and 10,234 genes were identified as ‘Class III: Lean_Ag-DEGs’ and ‘Class IV: Obese_Ag-DEGs’, respectively at Q < 0.01. We categorized these DEGs into four subcategories by considering the log_2_ fold change (*log*_*2*_*FC*) in gene expression along with the Q < 0.01 threshold (Additional file 8: Figure S5). Subsequently, for each of these categories, Class III and Class IV intersected, which led to three subcategories of DEGs, i.e., ‘lean AC adipogenesis-specific (LS)’, ‘obese AC adipogenesis-specific (OS)’, and ‘commonly altered for both (CA)’. Interestingly, most inflammatory genes, such as leukocyte migration, cell chemotaxis, and complement activation genes, were allocated in the highest upregulation category (Q < 0.01 and |*log*_*2*_*FC*|> 4) for both lean and obese AC adipogenesis (‘CA’) (Additional file 8: Figure S5B and Additional file 9: Table S4). Surprisingly, the magnitude of upregulation of these inflammatory genes in ‘LS’ was significantly higher than that in ‘OS’ (Additional file 8: Figure S5B), indicating that both lean and obese adipogenesis are coupled with increased expression of inflammatory genes and that lean adipogenesis, rather than obese adipogenesis, requires stronger upregulation of these inflammatory genes. Consistently, these inflammatory genes showed greater upregulation in ‘Lean_Ag-DEGs’ than in ‘Obese_Ag-DEGs’ (Fig. 6a). Particularly, we confirmed that the extent of upregulation of three genes including *IL-6, IL-1β, and TNF-α*, i.e., the three most studied inflammatory genes related to obesity, was significantly greater in ‘Lean_Ag-DEGs’ than in ‘Obese_Ag-DEGs’ (*P* < 0.01) (Fig. 6b). Certainly, these results explain why the expression levels of these genes in obese ACs were lower than those in lean ACs (Fig. 1).

## Discussion

Obesity is often characterized as a low-grade inflammatory disease in which an enhanced inflammatory response in AT and the serum of obese organisms has been well established [41]. We reached a contradictory conclusion regarding the up- or downregulation of inflammatory genes involved in obesity by estimating DEGs between lean AT and obese AT samples respectively derived from human lean and obese cohorts. We found that inflammatory genes such as *IL-6* and *TNF-α* were actually expressed at lower levels in obese ACs than in lean ACs, indicating that inflammatory genes are downregulated in the obese condition. In fact, contrary to what was conventionally believed, several recent studies have reported that obese ATs do not show increased expression of inflammatory genes, as described in "Background" section [27, 28]. In addition, studies addressing the possible enhancement of inflammatory genes by analyzing DEGs between obese ATs and lean ATs collected from obese and lean cohorts, respectively, are surprisingly rare.

These results led us to revisit the notion that the upregulation of inflammatory genes may be the primary cause of obesity. We thus designed another comparison of gene expression between ACs and preACs, i.e., a comparison that was expected to estimate gene expression differences that occur during adipogenesis, leading to Class III: Lean_Ag-DEGs for the lean condition and Class IV: Obese_Ag-DEGs for the obese condition. As shown in Fig. 6, we showed that ACs express higher levels of inflammatory genes than do preACs for both lean and obese conditions, indicating that both lean and obese adipogenesis require increased inflammatory response genes. Interestingly, we observed that the extent of enhancement of inflammatory genes in ACs compared with preACs was significantly higher for lean conditions than for obese conditions (Fig. 6a, b, Additional file 8: Figure S5). We think that this result clearly supports Asterholm et al.’s [22] view of the positive role of inflammation in fat deposition, i.e., an attenuated inflammatory response may be linked to more harmful fat deposition.

Unfortunately, all of the AC and preAC samples we used in this study were purified from ATs distantly residing in the tumors of cancer patients. It is possible that cancer could induce molecular changes in the residing AT, or vice versa [42], and the downregulated inflammatory genes in the obese condition could be a byproduct of complex interactions between cancer and the surrounding tumors. However, we think this is unlikely because the influence of cancer over changes in gene expression in AT between the lean and obese conditions are mutually offset in the gene expression profiles and would not be detected in the DEG analysis. Moreover, Haffa et al. [28] showed that the correlation between obesity and AT gene expression exceeds the potential impact of colorectal cancer or cancer therapy when investigating the expression of genes in SAT and VAT from 233 colorectal cancers.

We validated our work by comparing our findings with those of Ehrlund et al.’s study deposited in the GEO database (GSE80654), a study that was conducted based on an experimental design similar to that in the present work, i.e., a comparison of gene expression between lean and obese AC samples, although the AT was derived from SAT not from VAT [43]. As mentioned in "Background" section, transcriptome-based studies designed to directly compare gene expression between lean and obese AT or between lean and obese ACs are rare. In particular, studies based on transcriptome data generated from ACs and preACs derived from lean VAT are extremely rare. Ehrlund et al. reported a list of DEGs that were significantly altered in obese ACs compared with lean ACs purified from human healthy SAT samples [43]; in total, 24 upregulated DEGs and 64 downregulated DEGs were reported. Interestingly, we found significant overlap in the list of ‘LO-DEGs’ between our study and Ehrlund et al.’s study. For instance, genes, including *NQO1*, *VLDLR*, etc., were upregulated, whereas genes including *IL-6*, *MMP2*, and *CD44* (i.e., inflammatory response genes) were downregulated in obese ACs compared with those in lean ACs. Approximately 21.6% (19/88) overlapped with the same direction (i.e., up or down) between our study and Ehrlund et al.’s study. We think that this degree of overlap is quite remarkable, considering that we used an mRNA-Seq platform whereas they used a microarray platform to produce the transcriptome data, they used healthy SAT whereas we used VAT distantly surrounding cancer tumors, and because a significant gene expression difference has been reported between SAT and VAT [44, 45]. In addition, Xing et al. [30]’s RNA-Seq-based transcriptome study of pigs showed that pigs with thicker back fat (corresponding to obese AT in the present work) showed significant lower expression of inflammatory genes than pigs with thinner back fat (corresponding to lean AT in the present work), which is consistent with our observations in the present work that inflammatory genes are downregulated in obese ACs compared to lean ACs.

Another important but often forgotten aspect is that AT is a complex organ with a residing mixture of highly heterogeneous cell types, including macrophages, other immune cells, preACs, endothelial cells, and lipid-filled ACs [46–49]. Compositional changes in these cells are associated with obesity and construct unique microenvironments within AT, entailing the synthesis and turnover of ECM components that lead to changes in adiposity accompanying limited/excessive nutritional supply [50]. A key player in regulating AT inflammation is macrophage inflammation. The involvement of macrophages in AT associated with obesity is known as M1 macrophage polarization. Unfortunately, due to the lack of purified macrophages, we were unable to investigate whether macrophages have a major role in enhanced inflammation in obese AT. Several studies have already noted that the source of enhanced inflammatory cytokines such as *TNF-α* and *IL-6* is macrophages rather than ACs [51, 52]. Instead, in the present work, we suggest that preACs are partly responsible for the enhanced inflammatory responses in obese AT.

## Conclusions

Using transcriptome-based analyses of mRNA-Seq data derived from human purified ACs and preACs between lean and obese conditions, we found that the canonical obesity-related upregulated genes, particularly inflammatory response genes, were expressed at significantly lower levels in obese ACs than in lean ACs. Moreover, the levels of these classes of genes increased in both lean ACs and obese ACs compared to the respective lean preACs and obese preACs; however, the levels of enhancement of these genes were even greater for lean ACs than obese ACs. We believe our present work will help to resolve some of the unanswered questions regarding the molecular alterations that occur in lean and obese fat accumulation.

## Methods

### Preparation of transcriptomes derived from human AC and preAC samples

All the transcriptome samples used in the present work were produced by one of the out-sourced studies performed by the Korea National Institute of Health (KNIH). KNIH made efforts to collecting epigenomes and transcriptomes as a participating institute of the International Human Epigenome Consortium (IHEC), granting research funds to recruited research groups (selected by an evaluation process) for collecting tissue samples and their epigenome data. The collected data were also strictly regulated and distributed by the KNIH to the research groups who had proposed to analyze them after evaluation. We were one of the research groups that were selected to access and analyze the raw data in the KNIH server called the open access (OA) system under limited permission.

### Ethics statement

This study was performed in accordance with the principles of the Declaration of Helsinki and was approved by the Kangwon National University Hospital (Chuncheon, Korea) Institutional Review Board (IRB) (KWNUIRB-2017-11-003).

### Purification of AC and preAC samples

Retrieving VAT from healthy individuals is extremely difficult, and the purification of ACs or preACs from a small amount of lean VAT is even more difficult; therefore, ACs and preACs were purified from AT that was isolated from VAT in the abdominal region during the surgical resection of human cancer patients. To exclude a possibility that gene expression in AT can be affected by tumors residing in locations distant from the AT we collected, we first confirmed that there was no cancer type bias between the lean and obese samples (Additional file 10: Table S5). Second, we confirmed that the CRP levels, i.e., an indicator of systemic inflammation, of the samples that we analyzed were all less than 1 except for one outlier (#ob50), indicating that no systemic cachexia response affected gene expression for both the lean and obese AT (or ACs/preACs) from the tumors (Additional file 11: Table S6). In addition, we found no bias in sex, age, or cancer grade between the lean and obese samples (Additional file 11: Table S6).

Then, the removed AT with blood vessels and connective tissue was washed with PBS to remove the blood, including white and red blood cells. The 100 ~ 200 g of fat that was collected from each patient was minced and treated with collagenase I for 1 h at 37 °C, in which the samples were washed three times with PBS every 20 min. The digested samples were filtered with 350 μm mesh to remove undigested tissue. Fetal bovine serum (10%) was added to stop the collagenase I reaction.

After centrifugation at 400*g* for 10 min, the supernatant fraction, i.e., the fraction containing mature ACs, and the stromal vascular fraction (SVF) pellet were collected separately (Additional file 12: Figure S6). Mature ACs washed with PBS and medium were then used for RNA isolation. The SVF pellet was incubated in red blood cell lysis buffer for 15 min to remove red blood cells, and the SVF pellet was retrieved again after centrifugation. The cells in the SVF pellet were filtered through 100 μm mesh, and 40 μm nylon mesh was used for MACS/FACS sorting to purify preadipocytes (CD45 − /CD34 + /CD31 − cells) (Additional file 12: Figure S6) [53].

### RNA extraction

We tried to collect ACs and preACs as a pair of samples obtained from the ATs from the same individual, but the success rate of extracting preACs was not very high, which is why the number of preACs is significantly lower than that of ACs, as described below.

Total RNA from 27 AC and 21 preAC samples was extracted with an RNeasy Lipid tissue kit (Qiagen, Hilden, Germany) and RNeasy Micro Kit (Qiagen, Hilden, Germany) using the manufacturer’s recommendations. The AC samples comprised 12 L-AC and 15 O-AC samples, whereas the preAC samples consisted of 3 L-preAC and 18 O-preAC samples. Obesity among collected patient samples was diagnosed by BMI > 25 kg/m^2^ rather than BMI > 30 kg/m^2^. Based on a report from the Korea National Institute of Health, Koreans are particularly troubled by a higher incidence of metabolic diseases coupled with obesity, even though they have a BMI lower than the worldwide average. This is why Korean obesity is diagnosed as BMI > 25 kg/m_2_ rather than BMI > 30 kg/m_2_ [54, 55].

### cDNA library preparation

Construction of cDNA libraries and sequencing were conducted by two different protocols for the two different batches of samples collected at different times. First, for the samples, including L-AC samples (#5, 9, 29, 34, 35, 37, 38, 39, 40, 42, 43, 46), O-AC samples (#3, 11, 24, 25, 30, 32, 36, 41, 49, 50, 51), L-preAC samples (#2, 10, 13), and O-preAC samples (#4, 8, 12, 18, 19, 20, 21, 33, 53, 54), cDNA libraries were constructed by a TruSeq^®^ RNA Sample Preparation Guide (Illumina, San Diego, CA) with the purified RNAs. rRNAs were removed using a Ribo-zero-rRNA removal kit (Illumina). Quality control (QC) of the samples was then conducted with a Bioanalyzer (Agilent Technologies, Santa Clara, CA). Sequencing of each clone in the constructed cDNA library was performed with the HiSeq 2000 platform (Illumina, San Diego, CA). Second, for the samples including O-AC (#59, 62, 64, 65) and O-preAC (#55, 56, 57, 58, 60, 61, 63, 66) samples, cDNA libraries were constructed using a TruSeq Stranded mRNA Sample Preparation Guide. Unfortunately, different cDNA libraries constructed by these two different protocols severely affected the classification of samples; therefore, we decided to exclude all the cDNAs constructed at later stages from further analyses.

### mRNA-Seq analysis to obtain DEGs

The sequencing reads of the samples mentioned above were checked for quality by FastQC (https://www.bioinformatics.babraham.ac.uk/). The adaptor sequences and low-quality sequences were trimmed by Trimmomatic (v0.35) [56]. The reference genome fasta file (GRCh38/hg38) was indexed by STAR (v2.7.1a) using the ‘genomeGenerated’ option. Then, the trimmed reads were mapped using STAR (v2.7.1a) to the indexed reference genome [57]. Annotation was conducted using the ‘GTF’ file of ‘GENCODE Gene Set’ (release 30) (https://www.gencodegenes.org/). Mapped reads were quantified using ‘htseq-count’. The read counts were then normalized and compared between the control and case samples (e.g., ‘L-AC *vs*. O-AC’, ‘L-preAC *vs.* O-preAC’, etc.) using ‘DESeq2’ [58].

### Data analysis

All statistical analyses and plots were performed using R (v3.5.1) with the Bioconductor package (v3.8) [59]. GO analysis was performed using the DAVID tool [60]. GSEA was performed using GSEA software (v3.0) [61]. Furthermore, we used the ‘clusterprofiler’ package and ‘ggplot2’ function in R (v3.5.1) for additional GO analysis and plotting [62]. The network graph of genes was constructed using Cytoscape software (v3.7.1) [63]. Other batch jobs were performed with custom-built Python scripts (v3.6.8) (https://www.python.org/).

## Supplementary information

**Additional file 1: Figure S1.** Schematic of the identification of the four different classes of DEGs‘Class I: AC-DEGs’ are identified from O-AC expression divided by L-AC expression, ‘Class II: preAC-DEGs’ are from O-preAC expression divided by L-preAC expression, Class III: ‘Lean_Ag-DEGs’ are from L-AC expression divided by L-preAC expression, and Class IV: ‘Obese_Ag-DEGs’ are from O-AC expression divided by O-preAC expression. The statistical criteria for detecting each of the DEGs are explained in ditional file 2: Table S1. ‘e[]’ indicates the expression levels of genes within the bracket.

**Additional file 2: Table S1**. Statistical thresholds tested for selecting DEGs.

**Additional file 3: Table S2.** The sample information used for the present analysis.

**Additional file 4: Table S3.** Statistical thresholds used for testing to select three subcategories of AC-DEGs.

**Additional file 5: Figure S2.** GSEA analysis of LO-DEGs. Four selected gene sets from the GSEA analysis are presented here. (A) and (B) are the gene sets for inflammatory response and angiogenesis, respectively, showing significant enrichment in Le, and (C) and (D) are the gene sets for cellular respiration and cellular metabolism, respectively, showing significant enrichment in Oe. The left panels represent the graph of the enrichment score (ES) generated from the GSEA analysis, and the right panels are the heatmaps constructed by the gene sets with significant ES scores. The cyan and red bars on top of the heatmaps represent the ‘Le-AC’ and ‘Oe-AC’ samples, respectively.

**Additional file 6: Figure S3.** Schematic of the strategy for dividing the DEGs into eight subcategories‘LI-DEGs’ and ‘IO-DEGs’ are intersected. A gene is determined to be ‘progressive-up’ when the gene is upregulated both in ‘LI-DEGs’ and ‘IO-DEGs’. A gene is defined as ‘progressive-down’ when the gene is downregulated both in ‘LI-DEGs’ and ‘IO-DEGs’. A gene is ‘initial-up’ when the gene is upregulated in ‘LI-DEGs’ but not in ‘IO-DEGs’. A gene is ‘initial-down’ when the gene is downregulated in ‘LI-DEGs’ but not in ‘IO-DEGs’. A gene is ‘later-up’ when the gene is not upregulated in ‘LI-DEGs’ but is upregulated in ‘IO-DEGs’. A gene is ‘later-down’ when the gene is not downregulated in ‘LI-DEGs’ but is downregulated in ‘IO-DEGs. A gene is ‘up-to-down’ when the gene is upregulated in ‘LI-DEGs’ and downregulated in ‘IO-DEGs’ and ‘down-to-up’ when the reverse applies.

**Additional file 7: Figure S4.** PCA plot of the preAC-DEGs. The L-preAC (turquoise) and O-preAC (red) samples are dotted along the axis of the first two principal components (PC1 and PC2).

**Additional file 8: Figure S5**. Analysis of the functions of genes that are significantly altered between lean and obese adipogenesis. A. ‘Clusterprofile’ analysis of GO functional terms. Lean_Ag-DEGs and Obese_Ag-DEGs are intersected, leading to three subcategories: ‘LS’, ‘OS’, and ‘CA’ (see the main text). For LS, OS, and CA, DEGs are subdivided into upregulated (i.e., genes that are expressed at higher levels in ACs than in preACs) and downregulated genes (i.e., genes that are expressed at lower levels in ACs than in preACs). Upregulation and downregulation are further divided into four groups by considering the log2FC in gene expression along with the Q < 0.01 threshold. Functional enrichment of genes in each class is investigated and plotted by ‘Clusterprofiler’. Refer to the main text for the meaning of each colored box. B. Left: Box plot of expression levels of inflammatory genes in the ‘CA’ category. A total of 99 inflammatory genes were found by mapping these genes to the annotations on GeneCards (http://genecards.org). Box plots are constructed using the Log2FC values calculated for each of the 99 genes between the AC and preAC samples for the lean and obese conditions, respectively. Right: Square Venn diagram showing the numbers of pro-/anti-inflammatory genes. Statistical significance is estimated by Wilcoxon’s test.

**Additional file 9: Table S4. **List of highest upregulated inflammatory genes.

**Additional file 10: Table S5**. Clinical information of the samples.

**Additional file 11: Table S6.** Statistical summary of the clinical information.

**Additional file 12: Figure S6.** Schematic of the purification of AC and preAC cells from AT. Refer to the Materials and methods section for the detailed procedures depicted in this schematic.

## Data Availability

The raw RNA-seq data including deidentified clinical information used for this study are available upon request to the division of genome science at Korea national institute of health (KNIH) after evaluating the request by KNIH DAC (data access committee).

## Abbreviations

AT: Adipose tissue
AC: Adipocyte
preAC: Preadipocyte
DEG: Differentially expressed gene
BAT: Brown adipose tissue
SAT: Subcutaneous white adipose tissue
VAT: Visceral white adipose tissue
SVF: Stromal vascular fraction
L-AC: Lean adipocyte
L-preAC: Lean preadipocyte
O-AC: Obese adipocyte
O-preAC: Obese preadipocyte
QC: Quality control
GO: Gene ontology
GSEA: Gene set enrichment analysis
BMI: Body mass index
WC: Waist circumference
FPG: Fasting plasma glucose
C-pep: C-peptide
HDL: High-density lipoprotein
LDL: Low-density lipoprotein
LS: Lean adipogenesis-specific
OS: Obese adipogenesis-specific
CA: Commonly altered for both
DM: Diabetes mellitus
HTN: Hypertension
