# Supplementary material for: Putative positive role of inflammatory genes in fat deposition supported by altered gene expression in purified human adipocytes and preadipocytes from lean and obese adipose tissues
Source: J Transl Med. 2020 Nov 12;18:433. doi: 10.1186/s12967-020-02611-6 (PMC7664034; doi:10.1186/s12967-020-02611-6)
Supplement: Supplementary file 1 — Additional file 1: Figure S1. Schematic of the identification of the four different classes of DEGs‘Class I: AC-DEGs’ are identified from O-AC expression divided by L-AC expression, ‘Class II: preAC-DEGs’ are from O-preAC expression divided by L-preAC expression, Class III: ‘Lean_Ag-DEGs’ are from L-AC expression divided by L-preAC expression, and Class IV: ‘Obese_Ag-DEGs’ are from O-AC expression divided by O-preAC expression. The statistical criteria for detecting each of the DEGs are explained in ditional file 2: Table S1. ‘e[]’ indicates the expression levels of genes within the bracket. [file 12967_2020_2611_MOESM1_ESM.pdf]

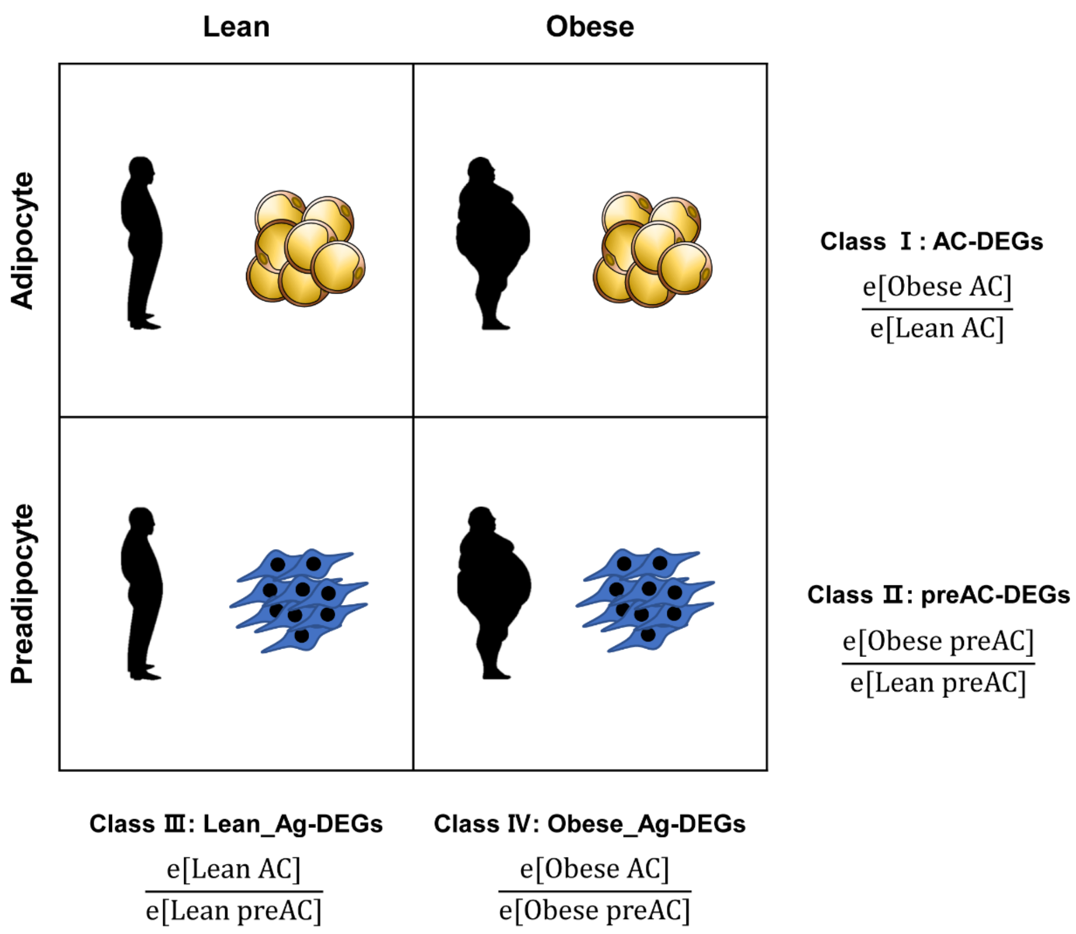

Figure S1. Schematic of the identification of the four different classes of DEGs

'Class I: AC-DEGs' are identified from O-AC expression divided by L-AC expression, 'Class II: preAC-DEGs' are from O-preAC expression divided by L-preAC expression, Class III: 'Lean_Ag-DEGs' are from L-AC expression divided by L-preAC expression, and Class IV: 'Obese_Ag-DEGs' are from O-AC expression divided by O-preAC expression. The statistical criteria for detecting each of the DEGs are explained in Supplementary Table 1. 'e[]' indicates the expression levels of genes within the bracket.
