# Supplementary material for: Putative positive role of inflammatory genes in fat deposition supported by altered gene expression in purified human adipocytes and preadipocytes from lean and obese adipose tissues
Source: J Transl Med. 2020 Nov 12;18:433. doi: 10.1186/s12967-020-02611-6 (PMC7664034; doi:10.1186/s12967-020-02611-6)
Supplement: Supplementary file 2 — Additional file 2: Table S1. Statistical thresholds tested for selecting DEGs. [file 12967_2020_2611_MOESM2_ESM.pdf]

Table S1. Statistical thresholds tested for selecting DEGs

Classes of DEGs	# of samples	Q < 0.01	Q < 0.05	P < 0.01	P < 0.05
Class I: AC-DEGs	11 O-AC/12 L-AC	66	314	1,198*	2,999
Class II: preAC-DEGs	10 O-preAC/3 L-preAC	0	2	213*	912
Class III: Lean_Ag-DEGs	8 L_e-AC/3 L-preAC	8,448*	10,007	9,124	10,729
Class IV: Obese_Ag-DEGs	7 O_e-AC/10 O-preAC	10,234*	11,561	10,724	12,062

‘*’ indicate the thresholds that were chosen for further analyses described in the main text.
