# Supplementary material for: Putative positive role of inflammatory genes in fat deposition supported by altered gene expression in purified human adipocytes and preadipocytes from lean and obese adipose tissues
Source: J Transl Med. 2020 Nov 12;18:433. doi: 10.1186/s12967-020-02611-6 (PMC7664034; doi:10.1186/s12967-020-02611-6)
Supplement: Supplementary file 3 — Additional file 3: Table S2. The sample information used for the present analysis. [file 12967_2020_2611_MOESM3_ESM.pdf]

Table S2. The sample information used for the present analysis

	Group	Samples
AC	L_e-AC	#5, #29, #34, #37, #38, #39, #43, #46
	I-AC	**#9**, <u>#24</u>, <u>#30</u>, **#35**, <u>#36</u>, **#40**, **#42**, <u>#51</u>
	O_e-AC	#3, #11, #25, #32, #41, #49, #50
preAC	L-preAC	#2, #10, #13
	O-preAC	#4, #8, #12, #18, #19, #20, #21, #33, #53, #54
Lean_ Ag-DEGs	L-preAC	#2, #10, #13
	L_e-AC	#5, #29, #34, #37, #38, #39, #43, #46
Obese_ Ag-DEGs	O-preAC	#4, #8, #12, #18, #19, #20, #21, #33, #53, #54
	O_e-AC	#3, #11, #25, #32, #41, #49, #50

Samples highlighted with the bold face and underline in the 'I-AC' category indicate the sample information that was originally assigned as lean and obese, respectively.
