# Supplementary material for: Putative positive role of inflammatory genes in fat deposition supported by altered gene expression in purified human adipocytes and preadipocytes from lean and obese adipose tissues
Source: J Transl Med. 2020 Nov 12;18:433. doi: 10.1186/s12967-020-02611-6 (PMC7664034; doi:10.1186/s12967-020-02611-6)
Supplement: Supplementary file 4 — Additional file 4: Table S3. Statistical thresholds used for testing to select three subcategories of AC-DEGs. [file 12967_2020_2611_MOESM4_ESM.pdf]

Table S3. Statistical thresholds used for testing to select three subcategories of AC-DEGs

Subcategories of AC-DEGs	Q < 0.01	Q < 0.05	P < 0.01	P < 0.05
LO-DEGs	2,657*	4,603	4,441	6,603
LI-DEGs	152	603	1,474*	3,318
O-DEGs	691	1,324*	1,877	3,469

‘*’ indicate the thresholds that were chosen for creating heatmaps and further analyses described in the main text.
