# Supplementary material for: Putative positive role of inflammatory genes in fat deposition supported by altered gene expression in purified human adipocytes and preadipocytes from lean and obese adipose tissues
Source: J Transl Med. 2020 Nov 12;18:433. doi: 10.1186/s12967-020-02611-6 (PMC7664034; doi:10.1186/s12967-020-02611-6)
Supplement: Supplementary file 5 — Additional file 5: Figure S2. GSEA analysis of LO-DEGs. Four selected gene sets from the GSEA analysis are presented here. (A) and (B) are the gene sets for inflammatory response and angiogenesis, respectively, showing significant enrichment in Le, and (C) and (D) are the gene sets for cellular respiration and cellular metabolism, respectively, showing significant enrichment in Oe. The left panels represent the graph of the enrichment score (ES) generated from the GSEA analysis, and the right panels are the heatmaps constructed by the gene sets with significant ES scores. The cyan and red bars on top of the heatmaps represent the ‘Le-AC’ and ‘Oe-AC’ samples, respectively. [file 12967_2020_2611_MOESM5_ESM.pdf]

A

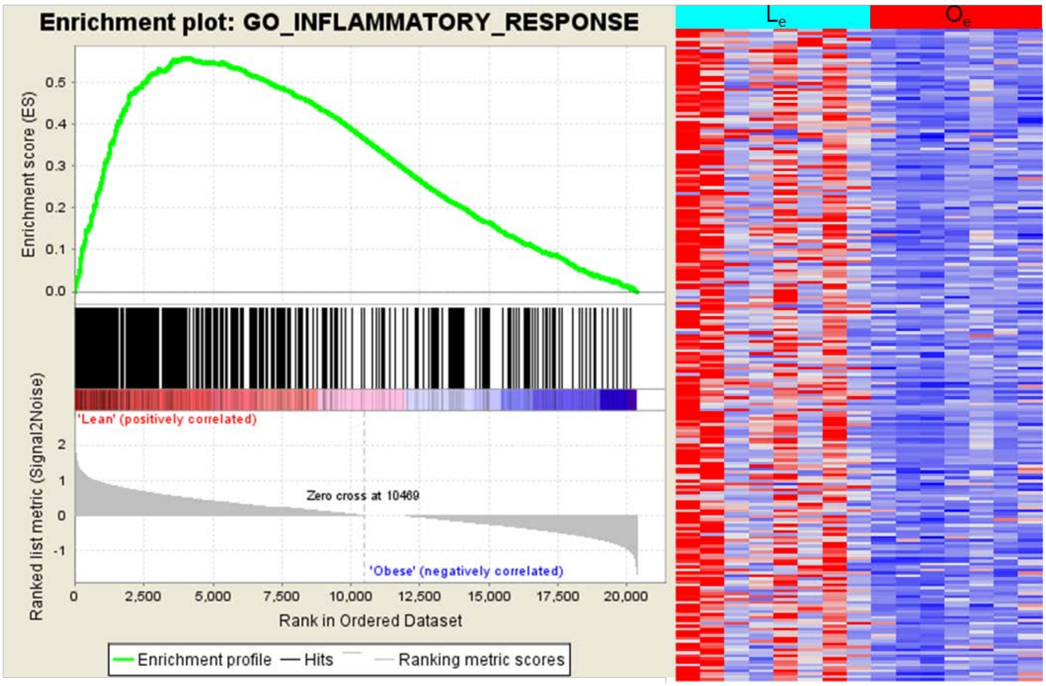

Enrichment plot: GO_INFLAMMATORY_RESPONSE
Enrichment score (ES)
0.5
0.4
0.3
0.2
0.1
0.0
'Lean' (positively correlated)
Ranked list metric (Signal2Noise)
2
1
0
-1
Zero cross at 10469
'Obese' (negatively correlated)
0
2,500
5,000
7,500
10,000
12,500
15,000
17,500
20,000
Rank in Ordered Dataset
Enrichment profile
Hits
Ranking metric scores
L_e
O_e

B

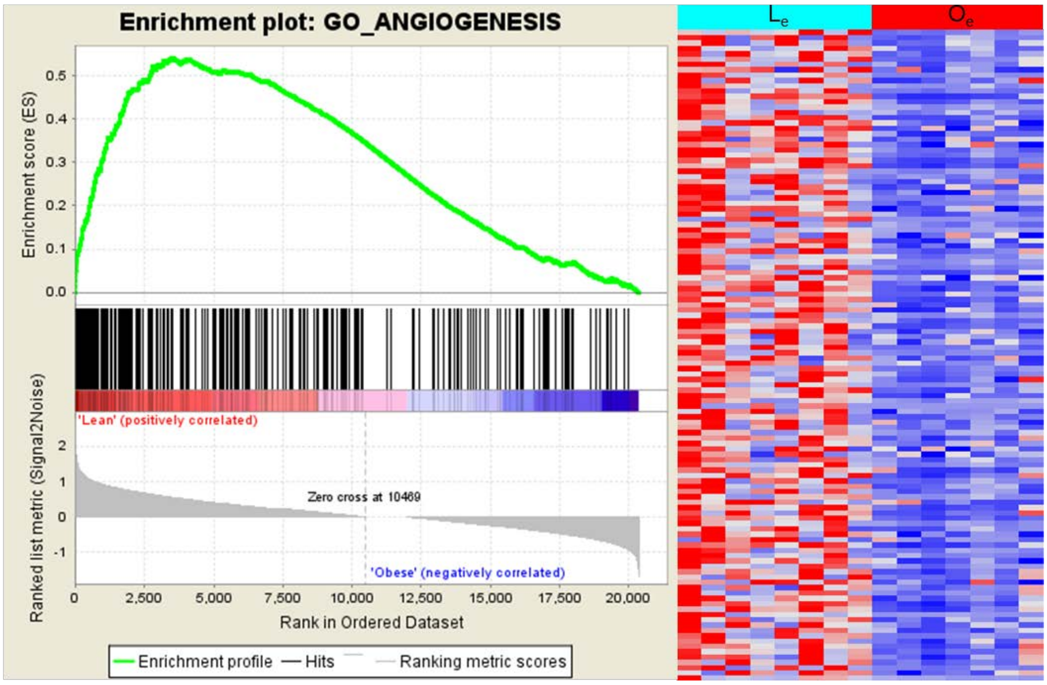

Enrichment plot: GO_ANGIOGENESIS
Enrichment score (ES)
0.5
0.4
0.3
0.2
0.1
0.0
'Lean' (positively correlated)
Ranked list metric (Signal2Noise)
2
1
0
-1
Zero cross at 10469
'Obese' (negatively correlated)
0
2,500
5,000
7,500
10,000
12,500
15,000
17,500
20,000
Rank in Ordered Dataset
Enrichment profile
Hits
Ranking metric scores
L_e
O_e

C

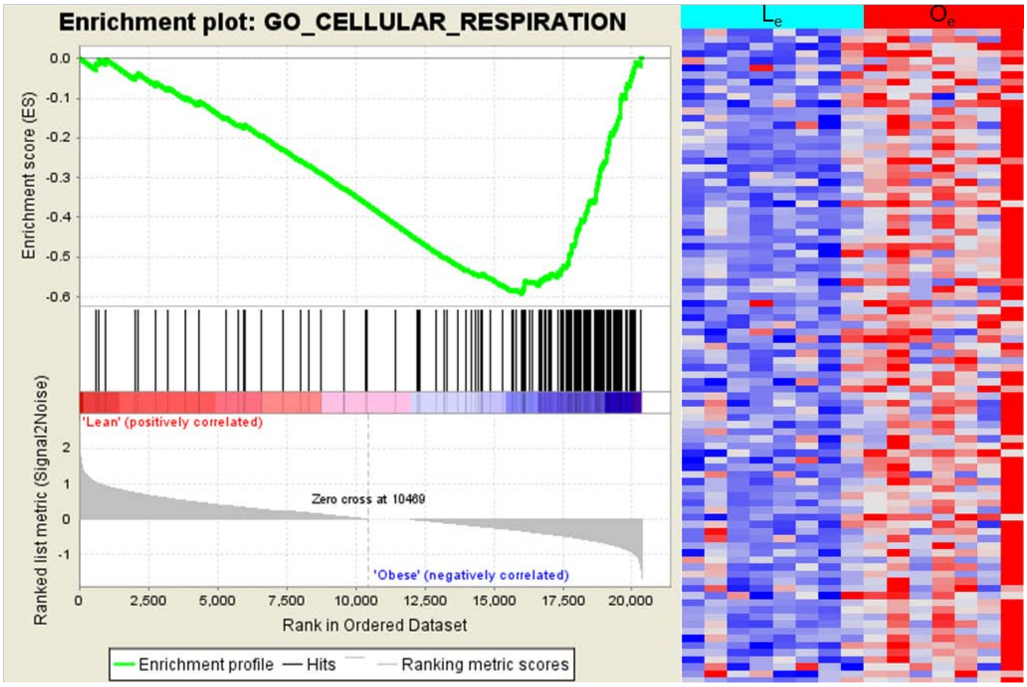

D

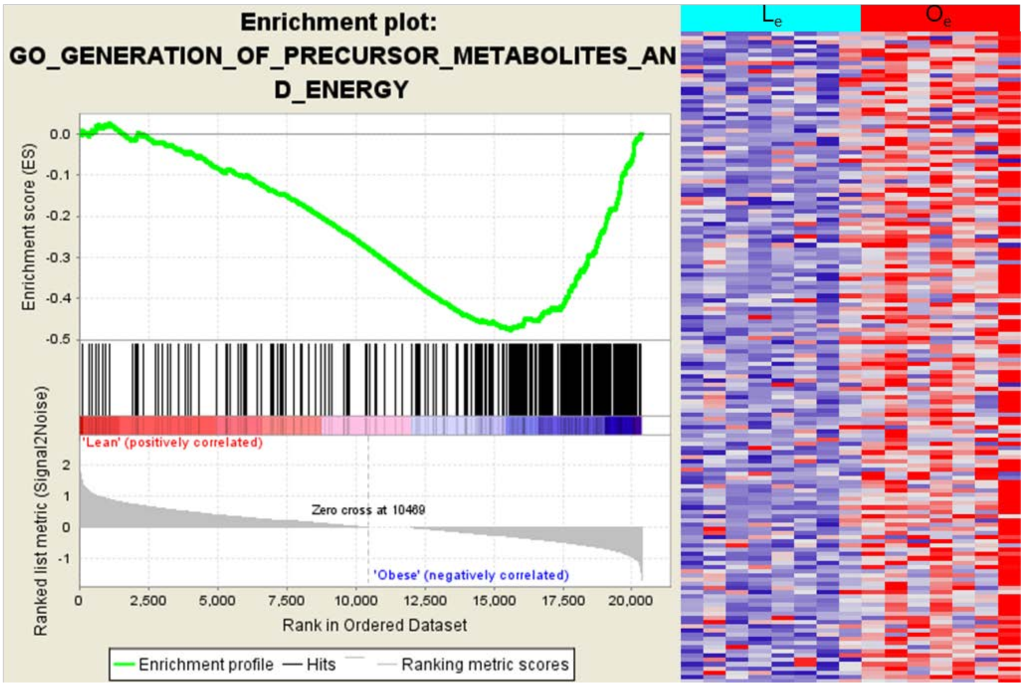

Figure S2. GSEA analysis of LO-DEGs

Four selected gene sets from the GSEA analysis are presented here. (A) and (B) are the gene sets for inflammatory response and angiogenesis, respectively, showing significant enrichment in L_e, and (C) and (D) are the gene sets for cellular respiration and cellular metabolism, respectively, showing significant enrichment in O_e. The left panels represent the graph of the enrichment score (ES) generated from the GSEA analysis, and the right panels are the heatmaps constructed by the gene sets with significant ES scores. The cyan and red bars on top of the heatmaps represent the 'L_e-AC' and 'O_e-AC' samples, respectively.
