# Supplementary material for: Putative positive role of inflammatory genes in fat deposition supported by altered gene expression in purified human adipocytes and preadipocytes from lean and obese adipose tissues
Source: J Transl Med. 2020 Nov 12;18:433. doi: 10.1186/s12967-020-02611-6 (PMC7664034; doi:10.1186/s12967-020-02611-6)
Supplement: Supplementary file 6 — Additional file 6: Figure S3. Schematic of the strategy for dividing the DEGs into eight subcategories‘LI-DEGs’ and ‘IO-DEGs’ are intersected. A gene is determined to be ‘progressive-up’ when the gene is upregulated both in ‘LI-DEGs’ and ‘IO-DEGs’. A gene is defined as ‘progressive-down’ when the gene is downregulated both in ‘LI-DEGs’ and ‘IO-DEGs’. A gene is ‘initial-up’ when the gene is upregulated in ‘LI-DEGs’ but not in ‘IO-DEGs’. A gene is ‘initial-down’ when the gene is downregulated in ‘LI-DEGs’ but not in ‘IO-DEGs’. A gene is ‘later-up’ when the gene is not upregulated in ‘LI-DEGs’ but is upregulated in ‘IO-DEGs’. A gene is ‘later-down’ when the gene is not downregulated in ‘LI-DEGs’ but is downregulated in ‘IO-DEGs. A gene is ‘up-to-down’ when the gene is upregulated in ‘LI-DEGs’ and downregulated in ‘IO-DEGs’ and ‘down-to-up’ when the reverse applies. [file 12967_2020_2611_MOESM6_ESM.pdf]

initial-up	initial-down	later-up	later-down
605	753	131	1,077

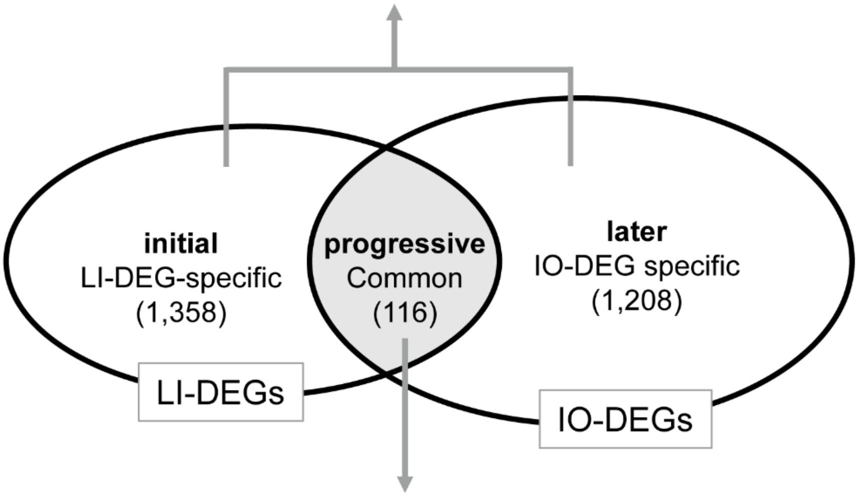

progressive-up (up-up)	progressive-down (down-down)	up-to-down	down-to-up
7	66	38	5

Figure S3. Schematic of the strategy for dividing the DEGs into eight subcategories

‘LI-DEGs’ and ‘IO-DEGs’ are intersected. A gene is determined to be ‘progressive-up’ when the gene is upregulated both in ‘LI-DEGs’ and ‘IO-DEGs’. A gene is defined as ‘progressive-down’ when the gene is downregulated both in ‘LI-DEGs’ and ‘IO-DEGs’. A gene is ‘initial-up’ when the gene is upregulated in ‘LI-DEGs’ but not in ‘IO-DEGs’. A gene is ‘initial-down’ when the gene is downregulated in ‘LI-DEGs’ but not in ‘IO-DEGs’. A gene is ‘later-up’ when the gene is not upregulated in ‘LI-DEGs’ but is upregulated in ‘IO-DEGs’. A gene is ‘later-down’ when the gene is not downregulated in ‘LI-DEGs’ but is downregulated in ‘IO-DEGs. A gene is ‘up-to-down’ when the gene is upregulated in ‘LI-DEGs’ and downregulated in ‘IO-DEGs’ and ‘down-to-up’ when the reverse applies.
