# Supplementary material for: Putative positive role of inflammatory genes in fat deposition supported by altered gene expression in purified human adipocytes and preadipocytes from lean and obese adipose tissues
Source: J Transl Med. 2020 Nov 12;18:433. doi: 10.1186/s12967-020-02611-6 (PMC7664034; doi:10.1186/s12967-020-02611-6)
Supplement: Supplementary file 7 — Additional file 7: Figure S4. PCA plot of the preAC-DEGs. The L-preAC (turquoise) and O-preAC (red) samples are dotted along the axis of the first two principal components (PC1 and PC2). [file 12967_2020_2611_MOESM7_ESM.pdf]

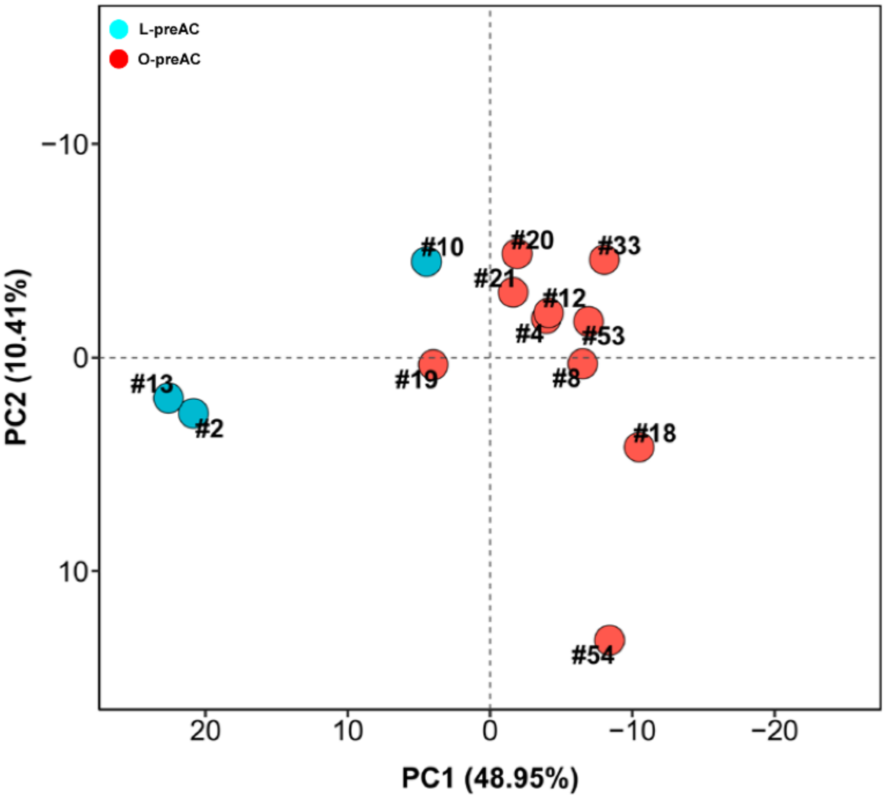

Figure S4. PCA plot of the preAC-DEGs

The L-preAC (turquoise) and O-preAC (red) samples are dotted along the axis of the first two principal components (PC1 and PC2).
