# Supplementary material for: Putative positive role of inflammatory genes in fat deposition supported by altered gene expression in purified human adipocytes and preadipocytes from lean and obese adipose tissues
Source: J Transl Med. 2020 Nov 12;18:433. doi: 10.1186/s12967-020-02611-6 (PMC7664034; doi:10.1186/s12967-020-02611-6)
Supplement: Supplementary file 8 — Additional file 8: Figure S5. Analysis of the functions of genes that are significantly altered between lean and obese adipogenesis. A. ‘Clusterprofile’ analysis of GO functional terms. Lean_Ag-DEGs and Obese_Ag-DEGs are intersected, leading to three subcategories: ‘LS’, ‘OS’, and ‘CA’ (see the main text). For LS, OS, and CA, DEGs are subdivided into upregulated (i.e., genes that are expressed at higher levels in ACs than in preACs) and downregulated genes (i.e., genes that are expressed at lower levels in ACs than in preACs). Upregulation and downregulation are further divided into four groups by considering the log2FC in gene expression along with the Q < 0.01 threshold. Functional enrichment of genes in each class is investigated and plotted by ‘Clusterprofiler’. Refer to the main text for the meaning of each colored box. B. Left: Box plot of expression levels of inflammatory genes in the ‘CA’ category. A total of 99 inflammatory genes were found by mapping these genes to the annotations on GeneCards (http://genecards.org). Box plots are constructed using the Log2FC values calculated for each of the 99 genes between the AC and preAC samples for the lean and obese conditions, respectively. Right: Square Venn diagram showing the numbers of pro-/anti-inflammatory genes. Statistical significance is estimated by Wilcoxon’s test. [file 12967_2020_2611_MOESM8_ESM.pdf]

A

B

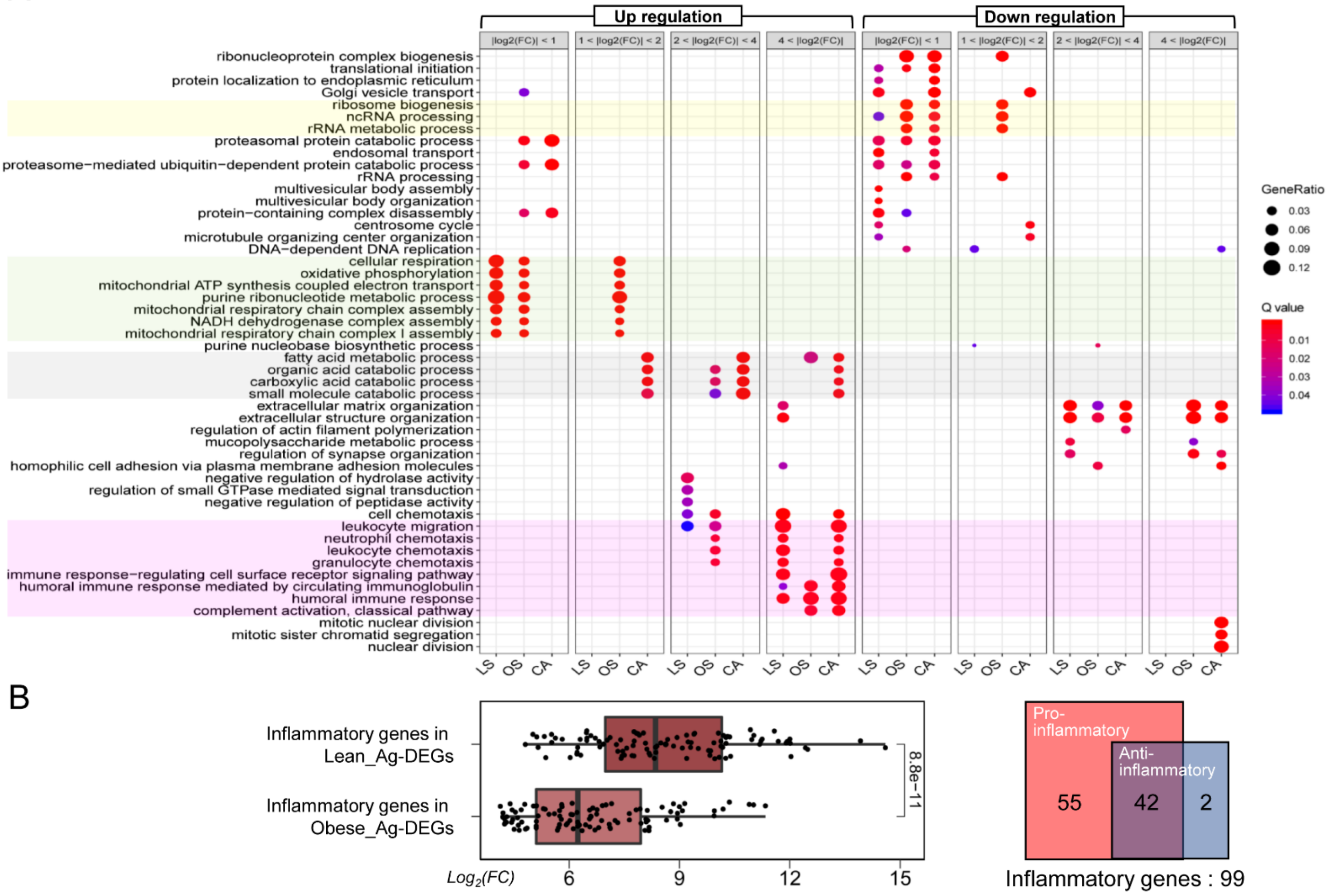

Figure S5. Analysis of the functions of genes that are significantly altered between lean and obese adipogenesis

A. ‘Clusterprofile’ analysis of GO functional terms. Lean_Ag-DEGs and Obese_Ag-DEGs are intersected, leading to three subcategories: ‘LS’, ‘OS’, and ‘CA’ (see the main text). For LS, OS, and CA, DEGs are subdivided into upregulated (i.e., genes that are expressed at higher levels in ACs than in preACs) and downregulated genes (i.e., genes that are expressed at lower levels in ACs than in preACs). Upregulation and downregulation are further divided into four groups by considering the log_2FC in gene expression along with the $Q < 0.01$ threshold. Functional enrichment of genes in each class is investigated and plotted by ‘Clusterprofiler’ (see Materials and methods). Refer to the main text for the meaning of each colored box. **B.** Left: Box plot of expression levels of inflammatory genes in the ‘CA’ category. A total of 99 inflammatory genes were found by mapping these genes to the annotations on GeneCards (http://genecards.org). Box plots are constructed using the Log_2FC values calculated for each of the 99 genes between the AC and preAC samples for the lean and obese conditions, respectively. Right: Square Venn diagram showing the numbers of pro-/anti-inflammatory genes. Statistical significance is estimated by Wilcoxon’s test.
