# Supplementary material for: Putative positive role of inflammatory genes in fat deposition supported by altered gene expression in purified human adipocytes and preadipocytes from lean and obese adipose tissues
Source: J Transl Med. 2020 Nov 12;18:433. doi: 10.1186/s12967-020-02611-6 (PMC7664034; doi:10.1186/s12967-020-02611-6)
Supplement: Supplementary file 11 — Additional file 11: Table S6. Statistical summary of the clinical information. [file 12967_2020_2611_MOESM11_ESM.pdf]

Table S6. Statistical summary of the clinical information

	Lean	Obese	*P*
**For Lean vs. Obese in AC**			
n	12	11	
Age (years)	64.2 ± 6.01[a]	61.1 ± 7.58	ns
Weight (kg)	58.8 ± 7.83	78.3 ± 7.52	**
BMI (kg/m^2)	22.5 ± 1.47	29.1 ± 3.23	**
FBS (mmol/L)	95 ± 19.8	122 ± 35.6	*
C-pep (ng/dL)	2.10 ± 1.64	2.86 ± 0.89	ns
HDL (mg/dL)	28.5 ± 10.5	31 ± 12	ns
LDL (mg/dL)	94.3 ± 32.5	82.4 ± 28.3	ns
CRP (mg/dL)	0.28 (0.14, 0.43)[b]	0.24 (0.1, 0.31)	ns
**For Lean vs. Obese in preAC**			
n	3	10	
Age (years)	70 (66, 71.5)	63.5 (59.8, 65.8)	ns
Weight (kg)	53.1 (44.7, 55.2)	76.8 (72.3, 86.7)	**
BMI (kg/m^2)	19.2 (15.8, 21.5)	29.5 (27.3, 31.2)	**
FBS (mmol/L)	100 (96.5, 104)	120 (104, 147)	ns
C-pep (ng/dL)	1.38 (1.13, 1.64)	2.1 (1.3, 3.4)	ns
HDL (mg/dL)	41.5 (38.3, 44.8)	33 (28, 39)	ns
LDL (mg/dL)	74 (66, 82)	109 (105, 123)	ns
CRP (mg/dL)	NA	NA	
**For L_e vs. O_e in AC**			
n	8	7	
Age (years)	63.7 ± 6.21	63.7 ± 4.79	ns
Weight (kg)	59.8 ± 6.78	79.7 ± 6.99	**
BMI (kg/m^2)	22.5 ± 1.56	29.5 ± 3.74	**
FBS (mmol/L)	102 ± 15.7	124 ± 26.1	ns
C-pep (ng/dL)	2.14 ± 1.94	2.82 ± 1.11	ns
HDL (mg/dL)	23.7 ± 8.91	25.8 ± 8.35	ns
LDL (mg/dL)	81.9 ± 23.4	86.6 ± 63.3	ns
CRP (mg/dL)	0.24 (0.14, 0.38)	0.1 (0.1, 0.24)	ns

a, standard deviation; b, inter quartile range; ns, no significance; ‘*’, $p < 0.05$; ‘**’, $p < 0.01$
