# Supplementary material for: Putative positive role of inflammatory genes in fat deposition supported by altered gene expression in purified human adipocytes and preadipocytes from lean and obese adipose tissues
Source: J Transl Med. 2020 Nov 12;18:433. doi: 10.1186/s12967-020-02611-6 (PMC7664034; doi:10.1186/s12967-020-02611-6)
Supplement: Supplementary file 12 — Additional file 12: Figure S6. Schematic of the purification of AC and preAC cells from AT. Refer to the Materials and methods section for the detailed procedures depicted in this schematic. [file 12967_2020_2611_MOESM12_ESM.pdf]

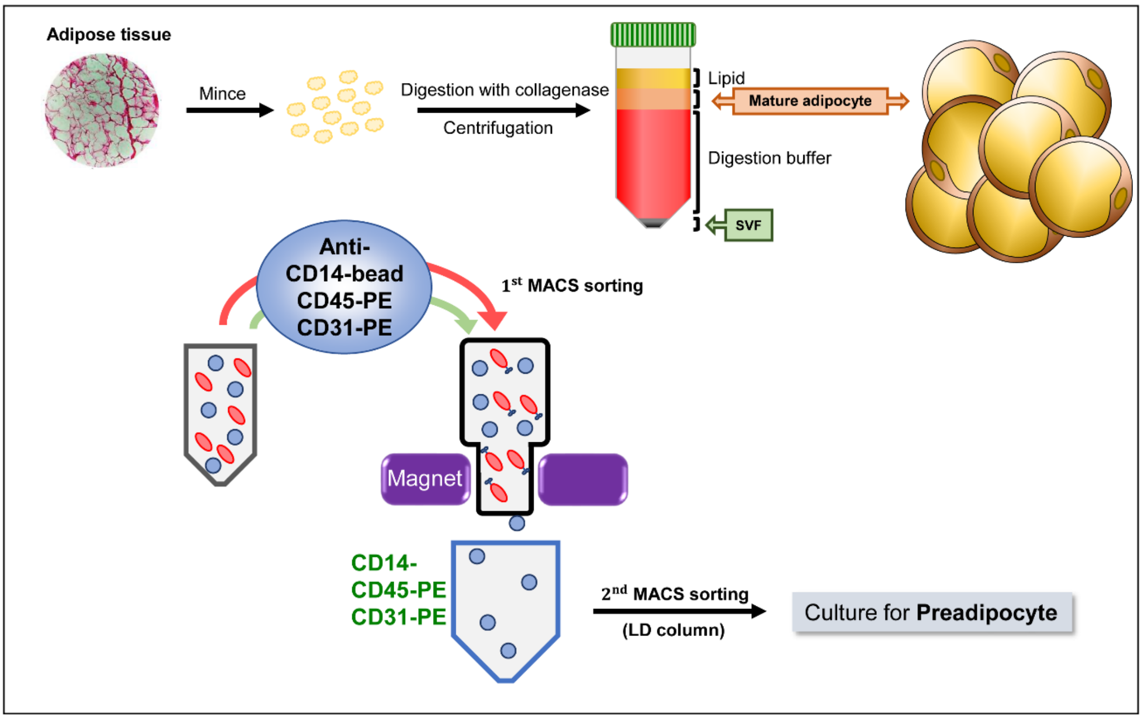

Figure S6. Schematic of the purification of AC and preAC cells from AT

Refer to the Materials and methods section for the detailed procedures depicted in this schematic.
